# Outcome assessment for Brachial Plexus birth injury. Results from the iPluto world‐wide consensus survey

**DOI:** 10.1002/jor.23901

**Published:** 2018-04-24

**Authors:** Willem Pondaag, Martijn J.A. Malessy

**Affiliations:** ^1^ Department of Neurosurgery Leiden University Medical Center Leiden The Netherlands

**Keywords:** peripheral nerve, spinal cord injury, spine

## Abstract

There is no consensus regarding strategies to optimally treat children with a brachial plexus birth injury (BPBI). Comparison of outcome data presented by different centers is impossible due to the use of (1) many different outcome measures to evaluate results; (2) different follow‐up periods after interventions; and (3) different patient ages at the time of assessment. The goal of iPluto (international PLexus oUtcome sTudy grOup) was to define a standardized dataset which should be minimally collected to evaluate upper limb function in children with BPBI. This dataset must enable comparison of the treatment results of different centers if prospectively used. Three rounds of internet surveys were used to reach consensus on the dataset. A Delphi‐derived technique was applied using a nine point Likert scale. Consensus was defined as having attained a rating of 7/8/9 by > = 75% of the participants. A total of 59 participants from five continents participated in the Second and Third Rounds of the survey. Consensus was reached regarding four elements: (1) evaluation should take place at the age of 1/3/5/7 years; range of motion in degrees should be measured for (2) passive joint movement; (3) active range of motion; and (4) the Mallet score should be determined. Consensus on how to asses and report outcome for BPBI was only reached on motor items from the “Body Function and Structure” domain. Consensus regarding additional ICF domains to obtain a more elaborate set of outcome items, should be addressed in future research. © 2018 The Authors. *Journal of Orthopaedic Research®* Published by Wiley Periodicals, Inc. on behalf of the Orthopaedic Research Society. J Orthop Res 36:2533–2541, 2018.

It is difficult to assess from the available literature which treatment strategy is optimal for children with severe brachial plexus birth injury (BPBI). In particular, there is no generally accepted algorithm to decide whether nerve surgery should be performed, and if so, at which age, and based on which parameters. Different algorithms are available, but these are usually based on relatively small patient series.^1^ The same holds true for other treatment modalities, such as tendon transfers, osteotomies, botulin toxin injection, casting, splinting, and physiotherapeutical exercises. Comparison and pooling of outcome data from different centers is currently virtually impossible because of the use of many different outcome measures, different follow‐up intervals and different patient ages. An attempt to pool data from different patient series showed resulting difficulties and inconsistencies.^2^ Two recent systematic reviews established that many different outcome measures have been used.^3^, ^4^

In an effort to improve comparability of research outcomes, the iPLUTO project, international PLexus oUtcome sTudy grOup, was initiated. The goal of iPluto was to define a universal dataset for the evaluation of upper limb function of children with BPBI. Ultimately, the prospective use of this dataset will enable comparison of published results and pooling of data. The latter is required, as the incidence of severe BPBI is less than 1/1,000 births.^5^ The intention of the iPluto project was not to set specific treatment guidelines or to interfere with current treatment policies. It would be a major step forward already to create an international standard how to evaluate and express results of treatment.

To obtain consensus we employed a Delphi survey, which is a well‐respected group facilitation technique. Its iterative multistage process was designed to transform opinion into group consensus.^6^ In short, questions are offered to participants of the surveys. After each round of replies, the groups responses are provided to the participants, hoping that the opinion of the group would—in subsequent rounds—shift towards consensus.^6^

In the current paper, the results of the first three rounds of the iPluto surveys are reported.

## METHODS

### Participant Recruitment

iPluto was announced at the XIX's International Symposium on Brachial Plexus Surgery (also known as the Narakas meeting) in February 2016, Barcelona. Attendees were requested to enlist online (http://iPluto.org). The email addresses were collected of attendees to the 2011 and 2016 Narakas meeting, and additionally attendees to the 2014 Toronto Obstetrical Brachial Plexus Palsy Workshop. The resulting email‐list consisted of 300 email‐addresses worldwide. The first email to announce the actual start of the project was sent on 29 April 2016. Recipients were encouraged to forward the iPluto announcement to other colleagues active in the field, with the request to register online.

Subsequent emails contained a personalized web to the internet questionnaire. The online questionnaire was designed with NetQuestionnaires, (Survalyzer Nederland B.V, Utrecht, The Netherlands, http://www.survalyzer.com/nl), responses were securely stored on the LUMC hospitals server.

### Rounds

The complete contents of the questionnaires are available as electronic appendices to this paper (Supplementary Appendix S1–S3).

The First Round consisted of inventory questions to inquire which methods the participants currently employ to assess outcome of children with BPBI. These were mostly binary questions (yes/no) to evaluate items that were extracted from the systematic review.^4^ In addition, participants were asked to add missing items to be included in subsequent rounds of the surveys. Additionally, details concerning the composition of their treatment team and annual patient load were asked.

A total of 300 invitations were sent by email; 20 email addresses proved to be outdated or false. The personalized weblink was activated by 107 people. Twenty‐seven responders did not answer a single question after clicking the hyperlink. Three entries were too incomplete to consider usable. On eight occasions, the survey was answered by two individuals from the same center. These double responses per center were not used, and the individual participants were notified by email to only provide one per center. Finally, the answers of 69 participants were analyzed in the First Round.

The software enabled participants to save their answers and continue later. The fastest time to answer the First Round was 5 min, median was 29 min. The longest interval between start and completion of the survey was 15 days.

The complete list of e‐mail addresses was used again to send an invitation for the Second Round, irrespective whether answers were received to the First Round. In the Second Round, participants had to score all items using a nine point Likert scale (one represented “fully disagree” and nine “fully agree”). During the analysis categories 7–9 were grouped as “in favour”/“agree,” categories 1–3 were grouped as “not in favour”/“disagree” and categories 4–6 were considered “neutral.”^7^ We defined consensus to have been reached if > = 75% of participants accepted (or rejected) a specific item.^6^ This scoring methodology had been revealed to the participants prior to the survey. Additionally, participants could add comments as free text to clarify their choice.

Identical scoring was repeated in the Third Round on the items for which consensus was not reached in the Second Round. The group's results of the Second Round were presented to the participants during the Third Round, as well as the provided free text comments. Only participants who answered to the Second Round, were invited to take part in the Third Round.

The First Round took place from June 2016 to September 2016, the Second Round from September 2016 to November 2016, and the Third Round from December 2016 to February 2017. An email reminder was sent after a few weeks if no response was received, or if the questionnaire was not finished completely. During the First and Second Round, two reminders were sent, during the Third Round four reminders were sent.

## RESULTS

### Participants and Practice Types

The final analysis only included the answers of participants for whom complete responses were available to both Rounds 2 and 3. Table 1 shows the number of complete responses for each round and combinations. In total 59 participants completed all questions in both Rounds 2 and 3, as shown (Table 1).

**Table 1:** Complete Responses (Marked as ‘+’) to Each Survey Round

*n*	Round 1	Round 2	Round 3
13	+	−	−
5	+	+	−
2	+	−	+
**49**	**+**	**+**	**+**
**10**	**−**	**+**	**+**
4	−	+	−
	69	68	61

The bold values represent that respondents were included in the final analysis.

**Figure 1:**
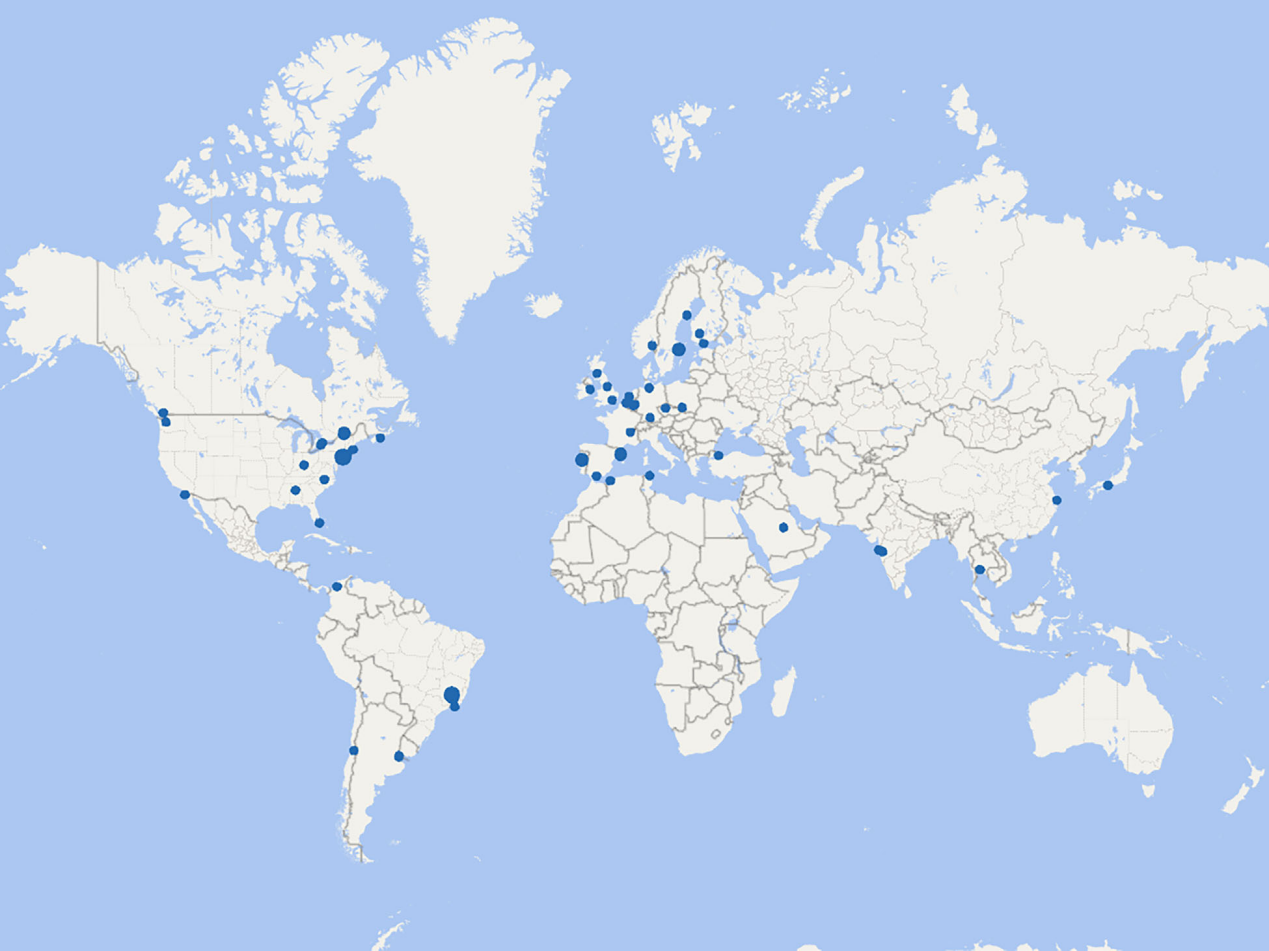
Contributing participants’ origin.

All but two respondents were surgeons. In Table 2 details regarding the practice of the participants are summarized. Most participants were based in Europe (42%) and North‐America (25%). The majority of respondents participated as member of a “brachial plexus team” (69%). Most practices received between 20 and 50 new patients each year. In Figure 1, the geographical locations of the participants’ practice are depicted to illustrate world‐wide participation.

**Table 2:** Details of 59 Participants

Continent			
Europe	25		
North‐America	15		
South‐America	10		
Asia	7		
Africa	2		
Specialty (self‐reported)			
Orthopedic surgeon	17		
Hand surgeon	14		
Plastic surgeon	9		
Neurosurgeon	8		
Nerve surgeon	5		
Rehabilitation specialist	1		
Child neurology	1		
Blank	4		
Practice type			
Team	41		
Solo	11		
Blank/other	7		
Team composition			
Occupational therapist	5		
Physical therapist	10		
Both	24		
None	20		
Annual caseload			
	New patients	Primary surgery	Secondary surgery
unknown/blank	4	4	4
<10	3	26	18
10–19	6	16	13
20–49	28	9	13
50–99	12	2	6
100‐	6	2	5

### Results of the Survey

In the Results section, the number of participants who agreed with the item (i.e., a score of 7, 8, or 9 on the 9‐point Likert scale) are presented in percentages.

### Evaluation of Lesion Severity

Consensus was reached concerning serial evaluation to take place at 1/3/6/9 months of age, which should include External rotation (measured in adduction), Abduction, Elbow flexion, Wrist extension, Finger flexion, and Finger extension.

The Narakas Classification^8^ was regarded suitable to express lesion severity (86%) and should be documented at 1 month of age (81%). There was consensus regarding the assessment of Elbow flexion, and Time to recovery of elbow flexion to express lesion severity. There was no consensus to express lesion severity with the use of Elbow strength (46%), the Toronto Test Score^9^ (37%), and the Cookie Test^10^ (20%).

There was consensus (78% of participants) regarding the number of root avulsions as appropriate assessment of lesion severity, but there was no consensus (63%) that the use of MRI or CT myelography is essential to analyze the presence of root avulsions.

### Timing of Evaluation

The proposal in the First Round was to evaluate at the age of 1/3/5/7 years, which was supported by 63/68 (93%) participants. Many participants suggested to add a time point at 2 years of age, and a time point as teenager, for example, at 15 years of age.

In subsequent rounds, consensus was reached to evaluate the child at the ages of 1/3/5/7 years. Consensus was nearly reached (74.6%) to evaluate the child additionally at 15 years of age.

### Treatment Outcome

Consensus was reached to include Passive Range of Motion in a minimal dataset (76%), regarding External rotation (measured in adduction), Abduction and Elbow extension.

Active Range of Motion expressed in degrees (AROM‐d) should also be included (95%), with evaluation of External rotation (measured in abduction and adduction), Abduction, Elbow flexion, Elbow extension, Wrist extension, Finger flexion, and Finger extension (Table 3).

**Table 3:** Results of the iPluto Survey Rounds 2 and 3 Concerning Items of Active Range of Motion in Degrees

	Score in Round 2			Score in Round 3		
Active range of motion in degrees (AROM‐d)	Mean	7–9	7–9 (%)	Mean	7–9	7–9 (%)
…is an appropriate outcome measure	8.76	58	**98**			
…is essential to be included in a minimal dataset	8.44	56	**95**			
Items						
External rotation (in abduction)	7.61	44	**75**			
External rotation (in adduction)	8.44	56	**95**			
Abduction	8.61	58	**98**			
Internal rotation	7.25	39	66	5.25	28	47
Elbow flexion	8.68	58	**98**			
Elbow extension	7.98	50	**85**			
Supination	7.49	43	73	6.46	37	63
Pronation	7.19	40	68	5.95	32	54
Wrist flexion	7.07	39	66	5.19	26	44
Wrist extension	7.98	55	**93**			
Finger flexion	7.90	49	**83**			
Finger extension	7.92	52	**88**			
Thumb flexion	7.29	41	69	4.97	24	41
Thumb extension	7.46	44	75	5.41	28	47

Results are shown as mean score of the 1–9 Likert scale, the total number of respondents scoring 7/8/9 and the related percentage of the total of 59 respondents. The Bold values represent items for which 75% consensus was reached.

The Mallet score^11^ was considered suitable as outcome measure (83%) and it should be expressed using sub scores (76%) for each movement. There was insufficient support for the use of aggregate scores (56%) or the use of the modified Mallet score^12^ (71%), which also includes “hand to belly” to assess active internal rotation.

No consensus was reached regarding Active Movement Scale (AMS)^13^ (61%), Force (MRC‐classification)^14^ (69%), Gilbert Shoulder Score (15%), Raimondi Hand Score^15^ (41%), Brachial Plexus Outcome Measure (BPOM)^16^ (39%), Assisting Hand Assessment (AHA)^17^ (29%), Semmes Weinstein filaments (31%), 2 point discrimination (29%), Pain Questionnaires (47%). Consensus was reached not to use the “nine hole peg test,”^18^ as 78% scored this as 1/2/3 (opposite consensus).

We asked participants if they had “sufficient experience with different PROMs to judge which PROMS are the most appropriate,” which was responded affirmatively by 19% of participants.

A comparison between the two largest contributing continents—Europe (25 participants) and North‐America (15 participants)—was made to detect preference differences regarding AROM‐d, AROM‐AMS, and MRC (Table 4). In both continents support for AROM‐d was >75%. In Europe more support was given to MRC grading, while in North‐America more support existed for AROM‐AMS. These differences were statistically significant in Round 2 (Table 4).

**Table 4:** Comparison Europe Versus North‐America

		Mean score		7‐9 (%)		
Items	Round	Europe	N‐America	Europe (%)	N‐America (%)	*p*
**AROM‐d**						
Appropriate	2	8.76	8.67	**100**	**93**	0.375
Inclusion	2	8.72	7.67	**100**	**80**	0.046*
**AROM‐AMS**						
Appropriate	2	6.44	7.53	48	**80**	0.046*
Inclusion	2	6.12	7.00	44	**80**	0.046*
Appropriate	3	6.44	7.73	60	**87**	0.152
Inclusion	3	6.32	7.40	60	**80**	0.298
**Strength (MRC)**						
Appropriate	2	7.20	5.73	**76**	47	0.089
Inclusion	2	7.16	4.87	**76**	40	0.042*
Appropriate	3	7.20	5.00	**76**	53	0.175
Inclusion	3	6.56	4.00	64	40	0.194

Active Range of Motion (AROM) in degrees versus AROM according to the Active Movement Scale, versus strength (Medical Resource Council). *p* values represent the outcome of Fisher's Exact Test. * = statistically significant. Bold values are >75%.

## DISCUSSION

This paper presents the results of the iPluto surveys (international PLexus oUtcome sTudy grOup) on how to measure and express severity, initial recovery, and treatment outcome in BPBI, either after conservative or surgical treatment. This is the first attempt to reach worldwide consensus on outcome measures for BPBI, with the aim to overcome the limitations of the current use of many different evaluation methods.^3^, ^4^ Different evaluation methods for children with BPBI have been summarized in a number of reviews in the recent years. AlQattan describes different methods to assess motor power.^19^ Details are described for the MRC, AMS, Mallet‐score, Gilbert shoulder score. Ho summarizes which methods are encountered in literature to evaluate both motor and sensory function in children with nerve lesions, and describes merits and drawbacks of different methods.^20^ Duff and DeMatteo provide a narrative review that describes evaluation methods that can be useful in the evaluation of children with a BPBI.^21^

**Figure 2:**
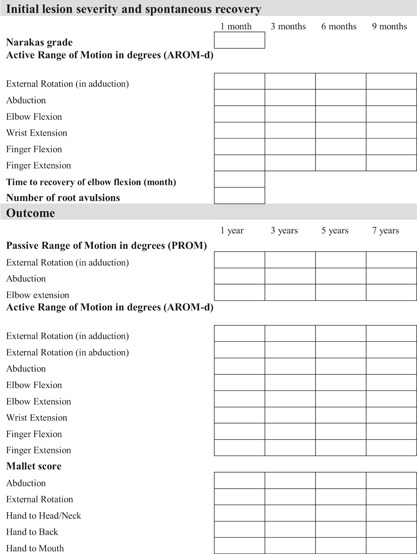
Template for a standardized score sheet to evaluate of BPBI patients.

The methods and outcomes of our Delphi surveys were reported in accordance to the standards set in a recent review.^22^ International participants reached consensus on a number of items, which we depicted in a template for use in the clinic (Fig. 2). The outcome of the survey was that initial lesion severity is adequately expressed using the Narakas classification,^8^ assessed at 1 month of age. Spontaneous recovery should be monitored at 1, 3, 6, and 9 months of age and expressed as degrees of active motion. Participants agreed that outcome should be assessed at fixed time points, namely at the age of 1, 3, 5, and 7 years. There was consensus that active range of motion in degrees (AROM‐d) and the Mallet scale should be employed as outcome measures.

Consensus was reached on a number of items which reflect motor evaluation of infants with a BPBI. If all reports on outcome of these children would include these outcome measures, patient series would be comparable and outcomes could be pooled for a meta‐analysis. As such, a large step forward would be made to decide which treatment strategies are optimal for these children. By no means the results of these surveys suggest that assessing and reporting only the proposed dataset is recommended. It is important to realize that “absence of consensus to include” does not mean there is “consensus not to include.” Every clinician and researcher should feel free to assess and report on the outcome of BPBI in their desired way, but is strongly encouraged to report at least the items from the minimal dataset to enable comparison with other studies. In the future, amendments and additions to this first dataset are possible. A first simple step that clinicians can take to comply with the iPluto system, is to organize their outpatient clinic in such a way that children are evaluated at the fixed time points that were decided on.

In the surveys, the AROM‐d had the largest support to express motor recovery evaluation. To measure AROM‐d has the advantages that it is intuitive, and it is a continuous scale. Normative values are readily available.^23^ The general method to measure and express range of motion has been defined in the past,^24^ but it is unclear from many papers how exactly the measurement took place. The use of a goniometer during the physical examination of children is cumbersome, but it provides more precise measurements than estimation. Without a goniometer, the stated number of degrees may suggest a higher precision than was obtained in reality. Especially in brachial plexus lesions, compensatory movements are frequent, and the examiner should take measures to avoid compensation.^25^ A consensus how to assess AROM‐d should be agreed upon. An additional drawback of AROM‐d is that it requires gravity to be eliminated before movement in the joint is noted. This implicates that when muscle contraction is present, but too weak to overcome gravity, the resulting degree measurement of movement remains zero.

Alternative assessments to AROM in degrees, are muscle force according to the MRC‐scale^14^ and AROM according to the Active Movement System (AMS),^13^ the latter has been developed in Toronto especially to evaluate children with BPBI. We compared support for these three systems across the continents of Europe and North‐America, and found that that the MRC‐scale was more supported in Europe, while the AMS‐methodology was preferred in North America. The most frequent free‐text comment during the survey rounds on the MRC was that it measures volitional muscle force. Obviously, babies or very young children will not respond to a request to execute a particular movement. An advantage of the MRC grading is that force can be quantified, although it is well known that the manual muscle testing may be subject to bias. Additionally, the range of motion is not included in the MRC systematics.

The largest advantage of the AMS system is that movements are scored during observation of spontaneous or elicited movements. As the AMS is a 7‐point grading system, extended statistical analysis is inherently possible.^13^ A downside of the system is that a later age, scores in the AMS lower than 4 do not correlate with clinically meaningful motor outcome levels. As such the AMS may be more useful to evaluate younger children, and less as an outcome parameter in older children. A hybrid system that combines both strength and range of motion has been proposed,^19^ but such a system has not been adopted by others. Participants did not rate such a hybrid system favorably during the Third Round of iPluto.

Participants chose the Mallet score next to AROM‐d to evaluate outcome. The Mallet score was initially developed as single score ranging from 0 to 5 based on five different movements,^11^ but many authors use the five consisting sub‐scores to separately evaluate specific movements. It was validated in 2003.^26^ A disadvantage of the Mallet system is that outcomes are usually clustered between scores 3 and 4, which reduces its capacity to discriminate, and thus may limit usefulness in statistical analysis. An advantage of the Mallet score is that the individual movements correspond to clinically relevant and intuitive situations. One of the subscores, for instance, evaluates the ability to bring the hand to the mouth. A recent publication pointed out that movement measured in AMS does not correlate well with Mallet scores.^27^ This finding supports measurement of both AROM and Mallet scores in children with BPBI.

Next to AROM and Mallet score, consensus was reached that passive ROM should be assessed and reported. This seems logical, as it is well known that children with BPBI develop contractures, most frequently internal rotation shoulder contractures and elbow flexion contractures.

Strong points of our survey are that a large number of respondents participated, based all over the world. Most participants were from Europe and North America. Unfortunately, no participants from Australia were recruited, probably because we employed attendee lists from an American and a European meeting. Two‐third of participants came from “brachial plexus centers.” We did not impose any specific criteria on participants, such as size of their patient population or years of experience. One may presume, however, that those attending a specialized brachial plexus meeting who in addition take the effort to complete the iPluto online surveys, have a certain commitment to and knowledge of BPBI.

We encountered a number of difficulties during the survey rounds. The first was a limited response rate. In the First Round we sent 280 emails to correct addresses, but we could only analyze 69 complete responses. It is probably not just, however, to calculate the response‐rate for the first round as low as 69/280 = 25% for a number of reasons. First, we targeted the audience of two brachial plexus meetings. One of these (the Narakas meeting) has a broader scope than BPBI only. The primary field of interest of some of the attendees may have been adult brachial plexus lesions, nerve compression syndromes or nerve tumors, and so they did not respond. Second, different members of the same center may have come to the meeting, and they choose one person to respond on behalf of the team.

Survey‐fatigue probably played an important role. Sending automated reminders to the participants proved powerful, as we saw that the first days after the reminder the number of contributions to the survey rose significantly. To achieve a proper response‐rate for the Third Round it proved necessary to send four reminders. For the Third Round 74 invitations were sent, which resulted in 63 usable responses. This results in a respectable response‐rate of 85% for the Third Round.

The second difficulty of the survey is that no differences were found between Round 2 and Round 3. This probably means that most participants stuck to their initial opinion, instead of adjusting their scoring on the basis of the groups opinion. Otherwise it could mean that many participants were satisfied with the outcomes of the second round.

The third difficulty was the limited variability in outcomes scores. Some of the proposed outcome measures concerned patient reported outcome measures (PROMs).^28^ Only 19% of participants judged themselves as capable to evaluate and rate different PROMs. Such unfamiliarity may result from the respondents’ background, as all but two respondents were surgeons. The intention of the iPluto survey— which had been advised as such during the survey—was that the survey questions were to be discussed within the respondent's brachial plexus team. We cannot judge in any way whether the surgeon‐respondent acted as spokesman for his team, or not. In many teams, an occupational therapist and/or a physical therapist take part, and these disciplines are eminently equipped for a broad view on how to assess outcome. The lack of support in the surveys for other ICF domains than motor outcome and lack of knowledge on PROMs make it questionable if elaborate discussion within the whole team actually took place in all cases.

As a result, unfortunately, the minimal dataset does not represent all domains of the World Health Organization's International Classification of Functioning, Disability and Health (WHO‐ICF).^29^ The current dataset only includes motor items in “Body Function and Structure.” In this respect, the initial goal of the iPluto surveys to reach consensus regarding all ICF domains was not met.

Items concerning sensory outcome and pain (from the domain “Body Function and Structure”) were not scored high enough to be included in the minimal dataset. Tools to evaluate other domains of the ICF are readily available and used in some clinics, but these were not ranked high enough in the current rounds by the iPluto participants. Available tools for evaluation of the domain “Activities and participation” are for instance the AHA/mini‐AHA,^17^ BPOM,^16^ Children's Hand‐use Experience Questionnaire (CHEQ),^30^ Disabilities of the Arm, Shoulder, and Hand (DASH) Outcome Measure,^31^, ^32^ Hand Use at Home (HUH)^33^ and Pediatric Outcomes Data Collection Instrument (PODCI).^26^, ^34^ For “Environmental Factors” the PedsQL Family impact module^35^ and PODCI^34^ are available.

Different aspects of diverse outcome measured were discussed with the ICF as starting point; it was concluded that evaluation focus may shift during the life of an infant with BPBI.^21^ In the infant age, the assessment focus should be on *impairment*, which gradually shifts to *activity* at school age and to *participation* during adolescence.

In a systematic review the psychometric properties (reliability, validity, and responsiveness) of BPBI outcome measures are discussed.^36^ Reliability is the extent to which a measurement is free from error. Validity is the extent to which an outcome measure evaluates a variable of interest. Responsiveness refers to the ability of an outcome measure to detect clinically meaningful changes over time. In this systematic review, the outcome measures which showed to have the most robust psychometric properties include the Active Movement Scale, the Assisting Hand Assessment, the PEDI, and the PODCI. Psychometric properties of outcome measures are important, but a specific test should not be discarded when the psychometric properties were not formally tested in the target population. Clinical utility—such as the administration, scoring, interpretation, and feasibility of using an outcome measure—are equally important when selecting an outcome measure for use in clinical and research settings.^36^ Practical and theoretical recommendations how to choose PROMs have recently been proposed.^28^

We hope to address a wider spectrum of outcome parameters in future research, in collaboration with clinicians and researchers worldwide. This may be done by sending out a new set of surveys, focusing on additional ICF domains, and including input from new participants. Alternatively,—or additionally—an international consensus meeting could be organized.

## CONCLUSION

This first world‐wide consensus survey on how to measure and report outcome in children with an BPBI provides the field with a minimal dataset for which consensus exists. All treating physicians should implement this dataset and timing protocol in their clinic and report all the elected items in scientific papers on outcome. This will result in increased comparability of papers, enables pooling of data, which eventually may lead to improved treatment strategies for children with a BPBI. This dataset should be used as a basis, and additional items from other ICF domains should be included in the future.

## AUTHORS’ CONTRIBUTIONS

Willem Pondaag and Martijn Malessy both contributed to research design. Willem Pondaag executed the surveys, and analyzed the results. Willem Pondaag drafted the manuscript, Martijn Malessy revised it critically. Both authors have read and approved the final submitted manuscript.

## Supporting information

Additional supporting information may be found in the online version of this article.

Supporting Appendix S1.

Supporting Appendix S2.

Supporting Appendix S3.
