# Supplementary material for: Outcome assessment for Brachial Plexus birth injury. Results from the iPluto world‐wide consensus survey
Source: J Orthop Res. 2018 Apr 24;36(9):2533–41. doi: 10.1002/jor.23901 (PMC6175006; doi:10.1002/jor.23901)
Supplement: Supplementary file 1 — Supporting Appendix S1. [file JOR-36-2533-s001.pdf]

iPluto - First Round

Welcome to the first internet survey from iPluto.
This first round of the internet surveys will use closed and open end questions to collect the methods that clinicians currently use to evaluate outcome in infants with a Neonatal Brachial Plexus Palsy / Obstetric Brachial Plexus Lesion. These methods are derived from literature and systematic reviews.

These methods (and new suggestions by respondents) will be rated in the iPluto rounds to follow, i.e. at this moment it is not necessary to express your *opinion* on the methods.

Use the [Back] and [Next] buttons on the bottom of the page to navigate, and press [Submit] on the last page. You can access the questionnaire only with the unique link sent to you by e-mail. With this link you can continue the questionnaire in case you aborted before completion of the survey.

Please note: For these Delphi surveys, one person will represent a brachial plexus center. Thereby, bias by vote overrepresentation from centers with many physicians will be avoided. It is assumed that this representative communicates the overall view that the specific center has.

On the iPluto website you will find the more detailed Project Outline.

Please provide information on your working environment.
We gather this information to link your responses to particular settings, also in later rounds.
Your answers will (of course) be processed anonymously.

Name (Last)	
Name (First)	
Department	
Hospital	
City	
Country	
What is your specialty	

What kind of practice do you have ?

- ○ solo-practice
- ○ brachial plexus team
- ○ other: ________

How many new NBPP patients do you see annually ? (average)	
How many NBPP patients are treated with nerve surgery annually ? (average)	
How many NBPP patients are treated with secondary surgery annually ? (average)	

The next questions apply when you work in a team.
How many people are in your team from each discipline ? (including yourself)

Orthopedic surgeon	
Neurosurgeon	
Plastic surgeon	
Microsurgeon	
Rehabilitation specialist	
Neurologist	
Physical therapist	
Occupational therapist	
Other (please [illegible]	

How do you currently evaluate the severity of the nerve lesion ?
(Not neccesarily for indication of nerve surgery only)

In your physical examination, do you regularly / routinely use...

Narakas classification ?	◯ Yes<br>◯ No
If YES, at which age(s) (months)	
Biceps strength ? (MRC)	◯ Yes<br>◯ No
If YES, at which age(s) (months)	
Active elbow flexion ? (either degrees or AMS)	◯ Yes<br>◯ No
If YES, at which age(s) (months)	
Toronto Test Score ?	◯ Yes<br>◯ No
If YES, at which age(s) (months)	
Cookie test ?	◯ Yes<br>◯ No
If YES, at which age(s) (months)	

Other items in physical examination to evaluate lesion severity ?
(please specify)

Which ancillary investigations do you currently employ to evaluate the severity of the nerve lesion ?
(Either for prognostication, for indication of nerve surgery, or for work-up for surgery)

Do you regularly / routinely use...

EMG: needle EMG ?	○ Yes<br>○ No
If YES, at which age(s) (months)	
EMG: conduction velocities ?	○ Yes<br>○ No
If YES, at which age(s) (months)	
CT-myelography ? (for root-avulsions)	○ Yes<br>○ No
If YES, at which age(s) (months)	
MRI ? (for root avulsions)	○ Yes<br>○ No
If YES, at which age(s) (months)	
Ultrasound of the brachial plexus ?	○ Yes<br>○ No
If YES, at which age(s) (months)	
Ultrasound of the diaphragm ?	○ Yes<br>○ No
If YES, at which age(s) (months)	

Other ancillary examinations ? (please specify)

How do you evaluate treatment outcome ?
(Either after surgery or after spontaneous recovery)

Do you regularly / routinely measure **passive range of motion** (in degrees) for...

Shoulder
- external rotation

○ Yes
○ No

If YES, how do you measure external rotation ?

☐ In adduction
☐ In abduction
☐ Both

Shoulder
- abduction

○ Yes
○ No

Shoulder
- internal rotation

○ Yes
○ No

Elbow
- flexion

○ Yes
○ No

Elbow
- extension

○ Yes
○ No

Elbow
- supination

○ Yes
○ No

Elbow
- pronation

○ Yes
○ No

Wrist
- flexion

○ Yes
○ No

Wrist
- extension

○ Yes
○ No

Finger
- flexion

○ Yes
○ No

Finger
- extension

○ Yes
○ No

Other PROM ?
(please specify)

How do you evaluate treatment outcome ?
(Either after surgery or after spontaneous recovery)

Do you regularly / routinely measure **active range of motion** (in degrees) for...

Shoulder<br>- external rotation	○ Yes<br>○ No

If YES, how do you measure external rotation ?

- ☐ In adduction
- ☐ In abduction
- ☐ Both

Shoulder<br>- abduction	○ Yes<br>○ No
Shoulder<br>- internal rotation	○ Yes<br>○ No
Elbow<br>- flexion	○ Yes<br>○ No
Elbow<br>- extension	○ Yes<br>○ No
Elbow<br>- supination	○ Yes<br>○ No
Elbow<br>- pronation	○ Yes<br>○ No
Wrist<br>- flexion	○ Yes<br>○ No
Wrist<br>- extension	○ Yes<br>○ No
Finger<br>- flexion	○ Yes<br>○ No
Finger<br>- extension	○ Yes<br>○ No

Other AROM ?
(please specify)

How do you evaluate treatment outcome ?
(Either after surgery or after spontaneous recovery)

Do you regularly / routinely measure **active range of motion** expressed according to the **AMS** (Active Movement Score from Toronto) for...

Shoulder<br>- external rotation	◯ Yes<br>◯ No
Shoulder<br>- abduction	◯ Yes<br>◯ No
Shoulder<br>- internal rotation	◯ Yes<br>◯ No
Elbow<br>- flexion	◯ Yes<br>◯ No
Elbow<br>- extension	◯ Yes<br>◯ No
Elbow<br>- supination	◯ Yes<br>◯ No
Elbow<br>- pronation	◯ Yes<br>◯ No
Wrist<br>- flexion	◯ Yes<br>◯ No
Wrist<br>- extension	◯ Yes<br>◯ No
Finger<br>- flexion	◯ Yes<br>◯ No
Finger<br>- extension	◯ Yes<br>◯ No

Other AMS-movements ?
(please specify)

How do you evaluate treatment outcome ?
(Either after surgery or after spontaneous recovery)

Do you regularly / routinely measure **force** **of these muscles (MRC grading)** for...

Shoulder abduction (deltoid muscle)	○ Yes<br>○ No
Elbow flexion (biceps muscle)	○ Yes<br>○ No
Elbow extension (triceps muscle)	○ Yes<br>○ No
Wrist extension	○ Yes<br>○ No
Grip strenght	○ Yes<br>○ No

Other MRC-scores ?
(please specify)

How do you evaluate treatment outcome ?
(Either after surgery or after spontaneous recovery)

Do you regularly / routinely employ **scoring systems** ?

Mallet-score	◯ Yes<br>◯ No

If YES, which version(s) of the Mallet do you employ ?

- ☐ each subscore (1-5 each)for abduction / external rot / hand-head / hand-mouth / hand-back
- ☐ including hand-belly (Modified Mallet Score)
- ☐ aggregate score (5-25)
- ☐ global score as originally described by Mallet (1-5)

Gilbert elbow-score	◯ Yes<br>◯ No
Raimondi hand-score	◯ Yes<br>◯ No
BPOM<br>(Brachial Plexus Outcome Measure)	◯ Yes<br>◯ No
AHA<br>(Assisting Hand Assessment)	◯ Yes<br>◯ No
Nine-Hole Peg Test	◯ Yes<br>◯ No

Other scoring systems ?

How do you evaluate treatment outcome ?
(Either after surgery or after spontaneous recovery)

Do you regularly / routinely assess **sensation and / or pain** using...

Testing touch qualitatively	○ Yes<br>○ No
Semmes Weinstein filaments	○ Yes<br>○ No
2 point discrimination	○ Yes<br>○ No
Pain questionnaire	○ Yes<br>○ No

Other methods for testing pain and / or sensation

How do you evaluate treatment outcome ?
(Either after surgery or after spontaneous recovery)

Do you regularly / routinely use **PROMs (Patient Reported Outcome Measures) / questionnaires** ...

PODCI<br>Pediatric Outcomes Data Collection Instrument	◯ Yes<br>◯ No
PEDI<br>Pediatric Evaluation of Disability Inventory	◯ Yes<br>◯ No
ABILHAND Manual Ability Measure	◯ Yes<br>◯ No
CHEQ Children's Hand-use Experience Questionnaire	◯ Yes<br>◯ No
HUH<br>Hand Use at Home	◯ Yes<br>◯ No
PedsQL<br>Family-Impact-Module	◯ Yes<br>◯ No
Functional Limb Preference Assessment	◯ Yes<br>◯ No

Other PROMs / questionnaires ?

The iPluto age proposal for timing of evaluation
Standardized time points for the collection of data should be used to compare results. iPluto proposes to use the age of the infant, and not the follow-up time after a specific intervention. The minimal number of evaluation moments will be limited to increase iPluto participation.
A first proposal would be to evaluate at the age of 1 / 3 / 5 / 7 years.

a. one year, because this is a plateau for spontaneous neurological recovery. Additionally, this time point may serve as a baseline before results of treatment interventions will have taken an effect.
b. three years, because this reflects the final stage of spontaneous recovery, and a plateau for shoulder function in children who were treated with early nerve reconstruction.
c. five years, because by this time an end-stage is reached for nerve reconstruction of the shoulder, and a plateau is reached for hand function. Additionally, it could serve as a pre-school assessment of function.
d. seven years, because by this time most secondary surgical procedures will have been performed and an end stage for hand function is reached. Limitations in the first year(s) of school and during leisure (e.g. sports) can be identified at this age because of sufficient cooperation.

This concept is in my opinion a ...

- ◯ Good idea
- ◯ Good idea, but... (please specify below)
- ◯ Bad idea, because... (please specify below)
- ◯ Don't know

If you have any comments, remarks or additions to this schedule please let us know below

The next questions are **not compulsory**.
In any case, please scroll down, click [Next] and [Submit] on the next page to complete the questionnaire.

These last 4 questions regard different items of the **ICF** (International Classification of Disease) domains which you judge to be relevant to patients with NBPP / OBPL.

Please list (separated by commas) **body functions** that are relevant and / or typical for patients with NBPP.
Body Functions are physiological functions of body systems (including psychological functions).

Please list (separated by commas) **body structures** that are relevant and / or typical for patients with NBPP.
Body Structures are anatomical parts of the body such as organs, limbs and their components.

Please list (separated by commas) **activities of daily living / participation** that are relevant and / or typical for patients with NBPP.
Activity is the execution of a task or action by an individual. Participation is involvement in a life situation.

Please list (separated by commas) **factors of the environment** that are relevant and / or typical for patients with NBPP.
Environmental Factors make up the physical, socal and attitudinal environment in which people live and conduct their lives.

Would you register to participate in the future development of a formal ICF Core Set for NBPP ?

- ○ Yes
- ○ No

ICF framework: (more information see WHO-website)

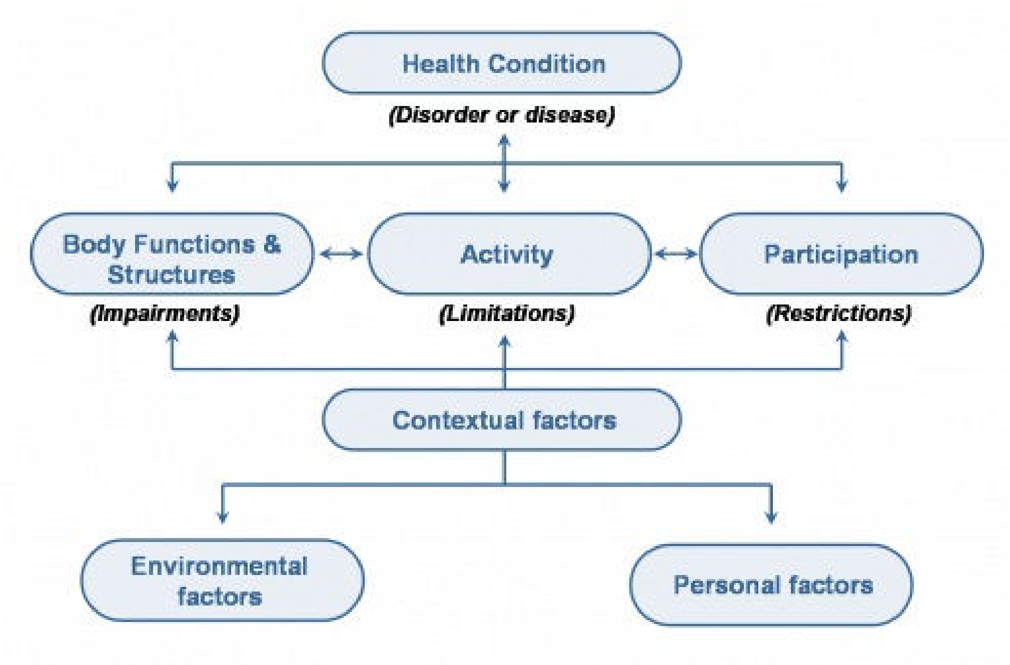

Thank you for your contribution.

You will automatically receive an invitation for the next round.

Please share any comments you have.
