# Supplementary material for: Outcome assessment for Brachial Plexus birth injury. Results from the iPluto world‐wide consensus survey
Source: J Orthop Res. 2018 Apr 24;36(9):2533–41. doi: 10.1002/jor.23901 (PMC6175006; doi:10.1002/jor.23901)
Supplement: Supplementary file 2 — Supporting Appendix S2. [file JOR-36-2533-s002.pdf]

Welcome to the second round of iPluto.

Please read the instructions below carefully.

The goal of the iPluto sequential questionnaires is consensus among participants on which items could serve as a minimal dataset for evaluation of outcome in OBPP infants.

Additionally it is important to agree how the severity of the lesion should be expressed, as it is necessary to document a baseline to stratify outcome depending on lesion severity.

In this round you will be asked to rate evaluation methods on a 1-9 Likert scale. In the subsequent questionnaire, the group response will be fed back to the participants, together with comments / arguments that can be typed as free text. The group feedback may change your appraisal of a specific item. In this way the aggregated opinion of participants will hopefully converge one way or another. When 75% of participants accept (or reject) an item, it is concluded that consensus is reached. Alternatively, the end result will be the percentage after 2-4 Delphi rounds. The number of Delphi rounds depends on abatement, as only participants who complete a survey will be invited for the subsequent round. We encourage participants to complete all rounds of the survey within the set time-frame, as the final evaluation will be determined in the last round. In this context, carefully select the one person from your center (when applicable) to serve as contact.

For the items you will be asked two separate questions, which – at first glance – may appear equal. Your are asked to rate whether a certain test is

a) appropriate to evaluate outcome, and
b) necessary to include in a minimal dataset.

The answer to these two questions may not necessarily be identical. A certain test may not be fully appropriate in your opinion, it may be necessary to include in the dataset, however, as no viable alternatives are available.

It is not the intention of the project to design a new evaluation method, but to choose the best items from available methods.

Importance of outcome categories

Please rate how important you judge that each outcome category is evaluated.

Passive range of motion (contractures)

	1	2	3	4	5	6	7	8	9	
Not at all important	( )	( )	( )	( )	( )	( )	( )	( )	( )	Most essential

Active range of motion

	1	2	3	4	5	6	7	8	9	
Not at all important	( )	( )	( )	( )	( )	( )	( )	( )	( )	Most essential

Muscle force

	1	2	3	4	5	6	7	8	9	
Not at all important	( )	( )	( )	( )	( )	( )	( )	( )	( )	Most essential

Sensation

	1	2	3	4	5	6	7	8	9	
Not at all important	( )	( )	( )	( )	( )	( )	( )	( )	( )	Most essential

Pain

	1	2	3	4	5	6	7	8	9	
Not at all important	( )	( )	( )	( )	( )	( )	( )	( )	( )	Most essential

Cosmesis / body image

	1	2	3	4	5	6	7	8	9	
Not at all important	( )	( )	( )	( )	( )	( )	( )	( )	( )	Most essential

Psychological functioning (anxiety, depression, loneliness)

	1	2	3	4	5	6	7	8	9	
Not at all important	( )	( )	( )	( )	( )	( )	( )	( )	( )	Most essential

Activities of daily living (eating, dressing, personal care)

	1	2	3	4	5	6	7	8	9	
Not at all	( )	( )	( )	( )	( )	( )	( )	( )	( )	Most

important										essential

School activities (writing)

	1	2	3	4	5	6	7	8	9	
Not at all important	( )	( )	( )	( )	( )	( )	( )	( )	( )	Most essential

Leisure activities (sports / music / hobbies)

	1	2	3	4	5	6	7	8	9	
Not at all important	( )	( )	( )	( )	( )	( )	( )	( )	( )	Most essential

Professional activities (paid work)

	1	2	3	4	5	6	7	8	9	
Not at all important	( )	( )	( )	( )	( )	( )	( )	( )	( )	Most essential

Support by parents

	1	2	3	4	5	6	7	8	9	
Not at all important	( )	( )	( )	( )	( )	( )	( )	( )	( )	Most essential

Interaction with peers / friends / classmates

	1	2	3	4	5	6	7	8	9	
Not at all important	( )	( )	( )	( )	( )	( )	( )	( )	( )	Most essential

Interaction with medical and paramedical specialists

	1	2	3	4	5	6	7	8	9	
Not at all important	( )	( )	( )	( )	( )	( )	( )	( )	( )	Most essential

Please provide your comments / extra outcome categories.
[____]
[____]
[____]
[____]

The following questions concern how the severity of the lesion should be expressed. It is necessary to document a baseline to stratify outcome depending on lesion severity.

How to evaluate the severity of the nerve lesion?
(Not necessarily for indication of nerve surgery only)

<u>Narakas classification</u>
The Narakas classification is used by 50/68 (74%) of respondents in the first round.

The Narakas classification is suitable to express initial lesion severity.
Please indicate your opinion…

	1	2	3	4	5	6	7	8	9	
fully disagree	( )	( )	( )	( )	( )	( )	( )	( )	( )	fully agree

The Narakas classification should be preferably assessed at
( ) 1 month of age
( ) 3 months of age
( ) 6 months of age
( ) 9 months of age
( ) serially at these time points
( ) never

Please provide your comments.
[____]
[____]
[____]
[____]

How to evaluate the severity of the nerve lesion?
(Not necessarily for indication of nerve surgery only)

<u>Biceps strength (MRC-grading)</u>
Biceps strength is used by 49/68 (72%) of respondents in the first round.

Testing biceps strength is suitable to express initial lesion severity.
Please indicate your opinion…

	1	2	3	4	5	6	7	8	9	
fully disagree	( )	( )	( )	( )	( )	( )	( )	( )	( )	fully agree

Biceps strength should be preferably assessed at
( ) 1 month of age
( ) 3 months of age
( ) 6 months of age
( ) 9 months of age
( ) serially at these time points
( ) never

Please provide your comments.
[____]
[____]
[____]
[____]

How to evaluate the severity of the nerve lesion?
(Not necessarily for indication of nerve surgery only)

Elbow flexion (movement)
Elbow flexion is used by 59/68 (87%) of respondents in the first round.

Testing elbow flexion is suitable to express severity of the lesion.
Please indicate your opinion…

	1	2	3	4	5	6	7	8	9	
fully disagree	( )	( )	( )	( )	( )	( )	( )	( )	( )	fully agree

Elbow flexion should be preferably assessed at
( ) 1 month of age
( ) 3 months of age
( ) 6 months of age
( ) 9 months of age
( ) serially at these time points
( ) never

Please provide your comments.
[____]
[____]
[____]
[____]

How to evaluate the severity of the nerve lesion?
(Not necessarily for indication of nerve surgery only)

Time to recovery of elbow flexion
Time to recovery (which month) of elbow flexion against gravity (i.e. MRC3 or better) was suggested by respondents in the first iPluto round.

Time to recovery of elbow flexion is suitable to express severity of the lesion.
Please indicate your opinion…

	1	2	3	4	5	6	7	8	9	
fully disagree	( )	( )	( )	( )	( )	( )	( )	( )	( )	fully agree

Please provide your comments.
[____]
[____]
[____]
[____]

How to evaluate the severity of the nerve lesion?
(Not necessarily for indication of nerve surgery only)

Which key-movements should be serially investigated at 1-3-6-9 months to globally express the severity of the lesion and the evolution of spontaneous recovery. Please try to limit the number of movements to increase completeness of the data, while keeping parameters complete for proper assessment.

Please indicate which active movements should be part of serial investigation.

	not necessary 1	2	3	4	5	6	7	8	Indispensable 9
External rotation (in abduction)	( )	( )	( )	( )	( )	( )	( )	( )	( )
External rotation (in adduction)	( )	( )	( )	( )	( )	( )	( )	( )	( )
Abduction	( )	( )	( )	( )	( )	( )	( )	( )	( )
Internal rotation	( )	( )	( )	( )	( )	( )	( )	( )	( )
Elbow flexion	( )	( )	( )	( )	( )	( )	( )	( )	( )
Elbow extension	( )	( )	( )	( )	( )	( )	( )	( )	( )
Supination	( )	( )	( )	( )	( )	( )	( )	( )	( )
Pronation	( )	( )	( )	( )	( )	( )	( )	( )	( )
Wrist flexion	( )	( )	( )	( )	( )	( )	( )	( )	( )
Wrist extension	( )	( )	( )	( )	( )	( )	( )	( )	( )
Finger flexion	( )	( )	( )	( )	( )	( )	( )	( )	( )
Finger extension	( )	( )	( )	( )	( )	( )	( )	( )	( )
Thumb flexion	( )	( )	( )	( )	( )	( )	( )	( )	( )
Thumb extension	( )	( )	( )	( )	( )	( )	( )	( )	( )

How to evaluate the severity of the nerve lesion?
(Not necessarily for indication of nerve surgery only)

Toronto Test score
The Toronto Test Score (aggregate of AMS score of elbow flexion and elbow, wrist, thumb, and finger extension) is used by 28/68 (41%) of respondents in the first round.

The Toronto Test Score is a suitable instrument to express initial severity of the lesion.
Please indicate your opinion…

	1	2	3	4	5	6	7	8	9	
fully disagree	( )	( )	( )	( )	( )	( )	( )	( )	( )	fully agree

The Toronto Test Score should be preferably assessed at
( ) 1 month of age
( ) 3 months of age
( ) 6 months of age
( ) 9 months of age
( ) never

Please provide your comments.
[____]
[____]
[____]
[____]

How to evaluate the severity of the nerve lesion?
(Not necessarily for indication of nerve surgery only)

Cookie Test
The Cookie Test is used by 42/68 (62%) of respondents in the first round.

The Cookie Test is a suitable instrument to express initial severity of the lesion.
Please indicate your opinion…

	1	2	3	4	5	6	7	8	9	
fully disagree	( )	( )	( )	( )	( )	( )	( )	( )	( )	fully agree

The Cookie Test should be preferably assessed at
( ) 1 month of age
( ) 3 months of age
( ) 6 months of age
( ) 9 months of age
( ) never

Please provide your comments.
[____]
[____]
[____]
[____]

How to evaluate the severity of the nerve lesion?
(Not necessarily for indication of nerve surgery only)

MRI is used by 41/69 (60%) of respondents in the first round to evaluate root avulsions, and 13/69 (13%) of respondents employed CT myelography.

MRI or CT-myelography is essential to assess the presence of root avulsions.
Please indicate your opinion…

	1	2	3	4	5	6	7	8	9	
fully disagree	( )	( )	( )	( )	( )	( )	( )	( )	( )	fully agree

The number of root avulsions is an appropriate way to express lesion severity.
Please indicate your opinion…

	1	2	3	4	5	6	7	8	9	
fully disagree	( )	( )	( )	( )	( )	( )	( )	( )	( )	fully agree

The following questions concern how to evaluate treatment outcome.

How to evaluate treatment outcome? - PROM
(Either after surgery or after spontaneous recovery)

Passive range of motion is used for specific movements by 85-95% of respondents in the first round.

Passive range of motion (in degrees) is an appropriate measure to express treatment outcome.
Please indicate your opinion…

	1	2	3	4	5	6	7	8	9	
fully disagree	( )	( )	( )	( )	( )	( )	( )	( )	( )	fully agree

Passive range of motion (in degrees) is essential to be included in a minimal dataset to publish or compare results.
Please indicate your opinion…

	1	2	3	4	5	6	7	8	9	
fully disagree	( )	( )	( )	( )	( )	( )	( )	( )	( )	fully agree

Please indicate for which movements it is necessary to evaluate passive range of motion.

	not necessary 1	2	3	4	5	6	7	8	Indispensable 9
External rotation (in abduction)	( )	( )	( )	( )	( )	( )	( )	( )	( )
External rotation (in adduction)	( )	( )	( )	( )	( )	( )	( )	( )	( )
Abduction	( )	( )	( )	( )	( )	( )	( )	( )	( )
Internal rotation	( )	( )	( )	( )	( )	( )	( )	( )	( )
Elbow flexion	( )	( )	( )	( )	( )	( )	( )	( )	( )
Elbow extension	( )	( )	( )	( )	( )	( )	( )	( )	( )
Supination	( )	( )	( )	( )	( )	( )	( )	( )	( )
Pronation	( )	( )	( )	( )	( )	( )	( )	( )	( )
Wrist flexion	( )	( )	( )	( )	( )	( )	( )	( )	( )
Wrist extension	( )	( )	( )	( )	( )	( )	( )	( )	( )
Finger	( )	( )	( )	( )	( )	( )	( )	( )	( )

flexion									
Finger extension	( )	( )	( )	( )	( )	( )	( )	( )	( )
Thumb flexion	( )	( )	( )	( )	( )	( )	( )	( )	( )
Thumb extension	( )	( )	( )	( )	( )	( )	( )	( )	( )

Please provide your comments.
[____]
[____]
[____]
[____]

How to evaluate treatment outcome? – AROM in degrees
(Either after surgery or after spontaneous recovery)

Active range of motion (in **degrees**) is used for specific movements by 85-95% of respondents in the first round.

Active range of motion (in degrees) is an appropriate measure to express treatment outcome.
Please indicate your opinion…

	1	2	3	4	5	6	7	8	9	
fully disagree	( )	( )	( )	( )	( )	( )	( )	( )	( )	fully agree

Active range of motion (in degrees) is essential to be included in a minimal dataset to publish or compare results.
Please indicate your opinion…

	1	2	3	4	5	6	7	8	9	
fully disagree	( )	( )	( )	( )	( )	( )	( )	( )	( )	fully agree

Please indicate for which movements it is necessary to evaluate active range of motion (in **degrees**) as treatment outcome.

	not necessary 1	2	3	4	5	6	7	8	Indispensable 9
External rotation (in abduction)	( )	( )	( )	( )	( )	( )	( )	( )	( )
External rotation (in adduction)	( )	( )	( )	( )	( )	( )	( )	( )	( )
Abduction	( )	( )	( )	( )	( )	( )	( )	( )	( )
Internal rotation	( )	( )	( )	( )	( )	( )	( )	( )	( )
Elbow flexion	( )	( )	( )	( )	( )	( )	( )	( )	( )
Elbow extension	( )	( )	( )	( )	( )	( )	( )	( )	( )
Supination	( )	( )	( )	( )	( )	( )	( )	( )	( )
Pronation	( )	( )	( )	( )	( )	( )	( )	( )	( )
Wrist flexion	( )	( )	( )	( )	( )	( )	( )	( )	( )
Wrist extension	( )	( )	( )	( )	( )	( )	( )	( )	( )
Finger	( )	( )	( )	( )	( )	( )	( )	( )	( )

flexion									
Finger extension	( )	( )	( )	( )	( )	( )	( )	( )	( )
Thumb flexion	( )	( )	( )	( )	( )	( )	( )	( )	( )
Thumb extension	( )	( )	( )	( )	( )	( )	( )	( )	( )

Please provide your comments.
[____]
[____]
[____]
[____]

How to evaluate treatment outcome? – AROM in AMS
(Either after surgery or after spontaneous recovery)

Active range of motion measured according to the **Active Movement System** (which originated in Toronto) is used by 50-60% of respondents in the first round.

Active range of motion (expressed as AMS) is an appropriate measure to express treatment outcome.
Please indicate your opinion…

	1	2	3	4	5	6	7	8	9	
fully disagree	( )	( )	( )	( )	( )	( )	( )	( )	( )	fully agree

Active range of motion (expressed as AMS) is essential to be included in a minimal dataset to publish or compare results.
Please indicate your opinion…

	1	2	3	4	5	6	7	8	9	
fully disagree	( )	( )	( )	( )	( )	( )	( )	( )	( )	fully agree

Please indicate for which movements it is necessary to evaluate active range of motion (in **AMS**).

	not necessary 1	2	3	4	5	6	7	8	Indispensable 9
External rotation (in abduction)	( )	( )	( )	( )	( )	( )	( )	( )	( )
External rotation (in adduction)	( )	( )	( )	( )	( )	( )	( )	( )	( )
Abduction	( )	( )	( )	( )	( )	( )	( )	( )	( )
Internal rotation	( )	( )	( )	( )	( )	( )	( )	( )	( )
Elbow flexion	( )	( )	( )	( )	( )	( )	( )	( )	( )
Elbow extension	( )	( )	( )	( )	( )	( )	( )	( )	( )
Supination	( )	( )	( )	( )	( )	( )	( )	( )	( )
Pronation	( )	( )	( )	( )	( )	( )	( )	( )	( )
Wrist flexion	( )	( )	( )	( )	( )	( )	( )	( )	( )
Wrist extension	( )	( )	( )	( )	( )	( )	( )	( )	( )

Finger flexion	( )	( )	( )	( )	( )	( )	( )	( )	( )
Finger extension	( )	( )	( )	( )	( )	( )	( )	( )	( )
Thumb flexion	( )	( )	( )	( )	( )	( )	( )	( )	( )
Thumb extension	( )	( )	( )	( )	( )	( )	( )	( )	( )

Please provide your comments.
[____]
[____]
[____]
[____]

How to evaluate treatment outcome? - Muscle Force
(Either after surgery or after spontaneous recovery)

Muscle force (MRC-grading) is used by 55-80% of respondents in the first round (depending on the item).

Muscle force (MRC) is an appropriate measure to express treatment outcome.
Please indicate your opinion…

	1	2	3	4	5	6	7	8	9	
fully disagree	( )	( )	( )	( )	( )	( )	( )	( )	( )	fully agree

Muscle force (MRC) is essential to be included in a minimal dataset to publish or compare results.
Please indicate your opinion…

	1	2	3	4	5	6	7	8	9	
fully disagree	( )	( )	( )	( )	( )	( )	( )	( )	( )	fully agree

Please indicate for which muscles it is necessary to evaluate muscle force.

	not necessary 1	2	3	4	5	6	7	8	Indispensable 9
Deltoid muscle	( )	( )	( )	( )	( )	( )	( )	( )	( )
Biceps muscle	( )	( )	( )	( )	( )	( )	( )	( )	( )
Triceps muscle	( )	( )	( )	( )	( )	( )	( )	( )	( )
Wrist extensors	( )	( )	( )	( )	( )	( )	( )	( )	( )
Grip strength	( )	( )	( )	( )	( )	( )	( )	( )	( )

Please provide your comments.
[____]
[____]
[____]
[____]

How to evaluate treatment outcome? – Scoring systems
(Either after surgery or after spontaneous recovery)

The **Mallet Score** is used by 58/67 (87%) of respondents in the first round.

The **Mallet Score** is an appropriate measure to express treatment outcome.
Please indicate your opinion…

	1	2	3	4	5	6	7	8	9	
fully disagree	( )	( )	( )	( )	( )	( )	( )	( )	( )	fully agree

The **Mallet Score** is essential to be included in a minimal dataset to publish or compare results.
Please indicate your opinion…

	1	2	3	4	5	6	7	8	9	
fully disagree	( )	( )	( )	( )	( )	( )	( )	( )	( )	fully agree

Please indicate which way you prefer to use the Mallet-score

	not necessary 1	2	3	4	5	6	7	8	Indispensable 9
Each of 5 subscores (grade 1-5 each)	( )	( )	( )	( )	( )	( )	( )	( )	( )
Add item: hand to belly (Modified Mallet)	( )	( )	( )	( )	( )	( )	( )	( )	( )
Aggregate (sum score)	( )	( )	( )	( )	( )	( )	( )	( )	( )

Please provide your comments.
[_______________________________________]
[_______________________________________]
[_______________________________________]
[_______________________________________]

How to evaluate treatment outcome? – Scoring systems
(Either after surgery or after spontaneous recovery)

The **Gilbert Elbow Score** is used by 19/67 (28%) of respondents in the first round.

The **Gilbert Elbow Score** is an appropriate measure to express treatment outcome.
Please indicate your opinion…

	1	2	3	4	5	6	7	8	9	
fully disagree	( )	( )	( )	( )	( )	( )	( )	( )	( )	fully agree

The **Gilbert Elbow Score** is essential to be included in a minimal dataset to publish or compare results.
Please indicate your opinion…

	1	2	3	4	5	6	7	8	9	
fully disagree	( )	( )	( )	( )	( )	( )	( )	( )	( )	fully agree

Please provide your comments.
[____]
[____]
[____]
[____]

How to evaluate treatment outcome? – Scoring systems
(Either after surgery or after spontaneous recovery)

The **Raimondi Hand Score** is used by 26/67 (39%) of respondents in the first round.

The **Raimondi Hand Score** is an appropriate measure to express treatment outcome.
Please indicate your opinion…

	1	2	3	4	5	6	7	8	9	
fully disagree	( )	( )	( )	( )	( )	( )	( )	( )	( )	fully agree

The **Raimondi Hand Score** is essential to be included in a minimal dataset to publish or compare results.
Please indicate your opinion…

	1	2	3	4	5	6	7	8	9	
fully disagree	( )	( )	( )	( )	( )	( )	( )	( )	( )	fully agree

Please provide your comments.
[____]
[____]
[____]
[____]

How to evaluate treatment outcome? – Scoring systems
(Either after surgery or after spontaneous recovery)

The **Brachial Plexus Outcome Measure (BPOM)** is used by 9/67 (13%) of respondents in the first round.

The **BPOM** is an appropriate measure to express treatment outcome.
Please indicate your opinion…

	1	2	3	4	5	6	7	8	9	
fully disagree	( )	( )	( )	( )	( )	( )	( )	( )	( )	fully agree

The **BPOM** is essential to be included in a minimal dataset to publish or compare results.
Please indicate your opinion…

	1	2	3	4	5	6	7	8	9	
fully disagree	( )	( )	( )	( )	( )	( )	( )	( )	( )	fully agree

The BPOM should be preferably assessed at the age of (multiple answers possible)
[ ] 1 year
[ ] 2 years
[ ] 3 years
[ ] 5 years
[ ] 7 years
[ ] 15 years
[ ] never

Please provide your comments.
[____]
[____]
[____]
[____]

How to evaluate treatment outcome? – Scoring systems
(Either after surgery or after spontaneous recovery)

The **Assisting Hand Assessment (AHA)** is used by 16/67 (24%) of respondents in the first round.

The **AHA** is an appropriate measure to express treatment outcome.
Please indicate your opinion…

	1	2	3	4	5	6	7	8	9	
fully disagree	( )	( )	( )	( )	( )	( )	( )	( )	( )	fully agree

The **AHA** is essential to be included in a minimal dataset to publish or compare results.
Please indicate your opinion…

	1	2	3	4	5	6	7	8	9	
fully disagree	( )	( )	( )	( )	( )	( )	( )	( )	( )	fully agree

The AHA should be preferably assessed at the age of (multiple answers possible)
[ ] 1 year
[ ] 2 years
[ ] 3 years
[ ] 5 years
[ ] 7 years
[ ] 15 years
[ ] never

Please provide your comments.
[____]
[____]
[____]
[____]

How to evaluate treatment outcome? – Scoring systems
(Either after surgery or after spontaneous recovery)

The **Nine hole peg test** is used by 6/67 (9%) of respondents in the first round.

The **Nine hole peg test** is an appropriate measure to express treatment outcome.
Please indicate your opinion…

	1	2	3	4	5	6	7	8	9	
fully disagree	( )	( )	( )	( )	( )	( )	( )	( )	( )	fully agree

The **Nine hole peg test** is essential to be included in a minimal dataset to publish or compare results.
Please indicate your opinion…

	1	2	3	4	5	6	7	8	9	
fully disagree	( )	( )	( )	( )	( )	( )	( )	( )	( )	fully agree

The Nine hole peg test should be preferably assessed at the age of (multiple answers possible)
[ ] 1 year
[ ] 2 years
[ ] 3 years
[ ] 5 years
[ ] 7 years
[ ] 15 years
[ ] never

Please provide your comments.
[____]
[____]
[____]
[____]

How to evaluate treatment outcome? - Sensation
(Either after surgery or after spontaneous recovery)

Semmes Weinstein filaments (SWf) were reported to be used by 14/67 (21%) of respondents in the first round.

SWf is an appropriate measure to express treatment outcome.
Please indicate your opinion…

	1	2	3	4	5	6	7	8	9	
fully disagree	( )	( )	( )	( )	( )	( )	( )	( )	( )	fully agree

SWf is essential to be included in a minimal dataset to publish or compare results.
Please indicate your opinion…

	1	2	3	4	5	6	7	8	9	
fully disagree	( )	( )	( )	( )	( )	( )	( )	( )	( )	fully agree

SWfilaments should be preferably assessed at the age of (multiple answers possible)
[ ] 1 year
[ ] 2 years
[ ] 3 years
[ ] 5 years
[ ] 7 years
[ ] 15 years
[ ] never

Please provide your comments.
[____]
[____]
[____]
[____]

How to evaluate treatment outcome? - Sensation
(Either after surgery or after spontaneous recovery)

2 point discrimination (2PD) is used by 17/67 (25%) of respondents in the first round.

2PD is an appropriate measure to express treatment outcome.
Please indicate your opinion…

	1	2	3	4	5	6	7	8	9	
fully disagree	( )	( )	( )	( )	( )	( )	( )	( )	( )	fully agree

2PD is essential to be included in a minimal dataset to publish or compare results.
Please indicate your opinion…

	1	2	3	4	5	6	7	8	9	
fully disagree	( )	( )	( )	( )	( )	( )	( )	( )	( )	fully agree

2PD should be preferably assessed at the age of (multiple answers possible)
[ ] 1 year
[ ] 2 years
[ ] 3 years
[ ] 5 years
[ ] 7 years
[ ] 15 years
[ ] never

Please provide your comments.
[_______________________________________]
[_______________________________________]
[_______________________________________]
[_______________________________________]

How to evaluate treatment outcome? - Pain
(Either after surgery or after spontaneous recovery)

Pain questionnaires is used by 21/67 (31%) of respondents in the first round.

Pain questionnaires is an appropriate measure to express treatment outcome.
Please indicate your opinion…

	1	2	3	4	5	6	7	8	9	
fully disagree	( )	( )	( )	( )	( )	( )	( )	( )	( )	fully agree

Pain questionnaires is essential to be included in a minimal dataset to publish or compare results.
Please indicate your opinion…

	1	2	3	4	5	6	7	8	9	
fully disagree	( )	( )	( )	( )	( )	( )	( )	( )	( )	fully agree

Pain questionnaires should be preferably assessed at the age of (multiple answers possible)
[ ] 1 year
[ ] 2 years
[ ] 3 years
[ ] 5 years
[ ] 7 years
[ ] 15 years
[ ] never

Please provide your comments.
[_______________________________________]
[_______________________________________]
[_______________________________________]
[_______________________________________]

How to evaluate treatment outcome? – PROMs
(Either after surgery or after spontaneous recovery)

PROMs (Patient Reported Outcome Measures) were reported to be used by 13 % of respondents in the first round.

Assessment of PROMs (Patient Reported Outcome Measures) is an appropriate method to express treatment outcome.
Please indicate your opinion…

	1	2	3	4	5	6	7	8	9	
fully disagree	( )	( )	( )	( )	( )	( )	( )	( )	( )	fully agree

PROMs are essential to be included in a minimal dataset to publish or compare results.
Please indicate your opinion…

	1	2	3	4	5	6	7	8	9	
fully disagree	( )	( )	( )	( )	( )	( )	( )	( )	( )	fully agree

I have / Our center has sufficient experience with different PROMs to judge which PROMS are the most appropriate.
Please indicate your opinion…

	1	2	3	4	5	6	7	8	9	
fully disagree	( )	( )	( )	( )	( )	( )	( )	( )	( )	fully agree

Please indicate whether the mentioned PROMs are appropriate to express treatment outcome. I you have no experience with or knowledge of a specific test please rate as **0.**

	unknown 0	fully disagree 1	2	3	4	5	6	7	8	fully agree 9
PODCI	( )	( )	( )	( )	( )	( )	( )	( )	( )	( )
PEDI	( )	( )	( )	( )	( )	( )	( )	( )	( )	( )
ABILHAND	( )	( )	( )	( )	( )	( )	( )	( )	( )	( )
CHEQ	( )	( )	( )	( )	( )	( )	( )	( )	( )	( )
HUH	( )	( )	( )	( )	( )	( )	( )	( )	( )	( )
PedsQL-FIM	( )	( )	( )	( )	( )	( )	( )	( )	( )	( )
E Handedness I	( )	( )	( )	( )	( )	( )	( )	( )	( )	( )

PODCI - Pediatric Outcomes Data Collection Instrument
PEDI - Pediatric Evaluation of Disability Inventory
ABILHAND Manual Ability Measure
CHEQ - Children's Hand-use Experience Questionnaire
HUH - Hand Use at Home questionnaire
PedsQL -Family-Impact-Module
Edinburgh Handedness Inventory (Limb Preference Assessment)

Please provide your comments.
[____]
[____]
[____]
[____]

The iPluto age proposal for timing of evaluation

Standardized time points for the collection of data should be used to compare results. iPluto proposes to use the age of the infant, and not the follow-up time after a specific intervention. Our first proposal in the first round was to evaluate at the age of 1 / 3 / 5 / 7 years.
•one year, because this is a plateau for spontaneous neurological recovery. Additionally, this time point may serve as a baseline before results of treatment interventions will have taken an effect.
•three years, because this reflects the final stage of spontaneous recovery, and a plateau for shoulder function in children who were treated with early nerve reconstruction.
•five years, because by this time an end-stage is reached for nerve reconstruction of the shoulder, and a plateau is reached for hand function. Additionally, it could serve as a pre-school assessment of function.
•seven years, because by this time most secondary surgical procedures will have been performed and an end stage for hand function is reached. Limitations in the first year(s) of school and during leisure (e.g. sports) can be identified at this age because of sufficient cooperation.

In the first round 63/68 (93%) supported this concept. Many participants suggested to add a time point at 2 years of age, and one as teenager, e.g. at 15 years of age.

Please note that time points before the age of one year will be collected to serve as baseline to express lesion severity.

Please indicate at which age it is desired to evaluate outcome.

	not necessary 1	2	3	4	5	6	7	8	Indispensable 9
1 year	( )	( )	( )	( )	( )	( )	( )	( )	( )
2 year	( )	( )	( )	( )	( )	( )	( )	( )	( )
3 year	( )	( )	( )	( )	( )	( )	( )	( )	( )
5 year	( )	( )	( )	( )	( )	( )	( )	( )	( )
7 year	( )	( )	( )	( )	( )	( )	( )	( )	( )
15 year	( )	( )	( )	( )	( )	( )	( )	( )	( )

Please provide your comments.
[____]
[____]
[____]

[____________________________________]
