# Supplementary material for: Outcome assessment for Brachial Plexus birth injury. Results from the iPluto world‐wide consensus survey
Source: J Orthop Res. 2018 Apr 24;36(9):2533–41. doi: 10.1002/jor.23901 (PMC6175006; doi:10.1002/jor.23901)
Supplement: Supplementary file 3 — Supporting Appendix S3. [file JOR-36-2533-s003.pdf]

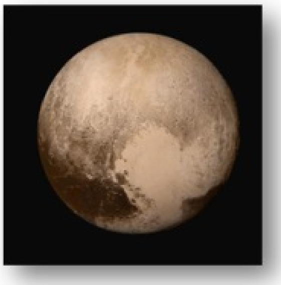

iPLUTO
international PLexus oUtcome sTudy grOup

Welcome to the third round of iPluto.

In this round you will be asked to rate the same evaluation methods from the previous round again on the 1-9 Likert scale. The group response from the previous round will be shown to you, together with comments / arguments that were entered. (Apologies for the technical issue that limited the length of this text for some of you.) The group feedback may change your appraisal of a specific item.

To summarize the group feedback from the previous round, the categories 7-9 are added up as 'in favour' / 'agree', categories 1-3 are added up as 'not in favour' / 'disagree' and categories 4-6 are considered 'neutral'.
We hope that your answers in this round will be directed towards 'in favour' or 'not in favour', so please avoid the neutral zone for answers, except of course if you really don't know which end to choose.

When 75% of participants accept (or reject) an item, it is concluded that consensus is reached. The number of questions will be smaller dan in the second round as consensus was reached on a few items in the previous round. The results from the previous round is presented in blue, the questions in black. As more text is presented for presentation of results, please view the survey on a large computer screen to see all content.

We encourage participants to complete all rounds of the survey within the set time-frame, as the final evaluation will be determined in the last round.

For the items you will be asked again two separate questions, which - at first glance - may appear equal. Your are asked to rate whether a certain test is
a) appropriate to evaluate outcome, and
b) necessary to include in a minimal dataset.
The answer to these two questions may not necessarily be identical. A certain test may not be fully appropriate in your opinion, it may be necessary to include in the dataset, however, as no viable alternatives are available. Otherwise, a test may be very good in determining outcome, but not useful in daily practice because it will take too much time or resources.

Summary of the previous round

Trying to summarize results may not be suitable already after the first round, but the following short summary may guide you through the survey.
Detailed results will be presented at each question.

To evaluate the severity of the lesion, there is consensus on the value of serial evaluation (at 1-3-6-9 months) of movement of external rotation, abduction, elbow flexion, wrist extension, finger flexion and finger extension are useful. Many support the Narakas Grade as summary of the clinical picture, but not sufficient for consensus. Time to recovery of elbow flexion against gravity was rated as useful to express lesion severity.

To evaluate outcome, there is consensus that joint restrictions of passive movement of external rotation, abduction and elbow extension are important to score. Active range of motion (in degrees) was judged as essential for external rotation (both in abduction and adduction), abduction, elbow flexion and extension, wrist extension, finger flexion and extension.
There was support between 50-75% for motion expressed as the Active Movement System, but not sufficient for consensus.
Force was also valued between 50-75%, the only item that reached consensus was biceps force.
Different scoring systems were rated; for shoulder movements the summary of the Mallet score was valued as consensus.
Support for other scoring systems was < 75%. The nine hole peg test was rated as not useful by > 75%.

The following questions concern how the **severity of the lesion** should be expressed.
It is necessary to document a baseline to stratify outcome depending on lesion severity.

How to evaluate the severity of the nerve lesion?
(Not necessarily for indication of nerve surgery only)

Narakas classification
The Narakas classification was rated by 71% as a suitable measure to express lesion severity, which means a majority supports the Narakas Grade, but consensus (75%) was not reached. Please rate again.

The results from the previous round were as follows
The Narakas classification is suitable to express initial lesion severity.

rating	**1**	**2**	**3**	**4**	**5**	**6**	**7**	**8**	**9**	Total
n	2		1	1	12	5	16	18	17	72
%	2,8		1,4	1,4	16,7	6,9	22,2	25,0	23,6	100,0

summary

	Sum 1-2-3	Sum 4-5-6	Sum 7-8-9
%	4,2	25,0	**70,8**

Comments:
'as early as possible', 'as i am mostly performing an EMNG. This can be correctly supported by the EMNG', 'At 3 months false movements cloud it', 'Evaluation at later dates is irrelevant as spontaneous recovery confuses. In addition, a severe lesion would not be observed serially for so long.', 'How originally detected/3 weeks', 'I don't use this so disregard my answers', 'I preferred the Toronto classification', 'I suggest 6 weeks and 3 months', 'The Narakas Classification allows to stratify the types of injury and to correlate with prognosis', 'The Narakas classification is simple to use, and provides a valuable summary of the clinical picture.'

The Narakas classification is suitable to express initial lesion severity.
Please indicate your opinion…

	1	2	3	4	5	6	7	8	9	
fully disagree	○	○	○	○	○	○	○	○	○	fully agree

The timing of the Narakas classification was seldomly chosen at 6 or 9 months in the previous round. (Narakas originally described the classification based on the recovery after 2-3 weeks.)
Please provide your opinion to evaluate the Narakas Grade at 1 month and 3 months; you can score both time points as important, or differently.

The Narakas classification should be assessed at 1 month.
Please indicate your opinion…

	1	2	3	4	5	6	7	8	9	
fully disagree	○	○	○	○	○	○	○	○	○	fully agree

The Narakas classification should be assessed at 3 months
Please indicate your opinion…

	1	2	3	4	5	6	7	8	9	
fully disagree	○	○	○	○	○	○	○	○	○	fully agree

Please provide your comments.

How to evaluate the severity of the nerve lesion?
(Not necessarily for indication of nerve surgery only)

Biceps strength (MRC-grading)

The results of the previous round for biceps strength (MRC-score) were as follows.
Testing biceps strength is suitable to express initial lesion severity.

rating	**1**	**2**	**3**	**4**	**5**	**6**	**7**	**8**	**9**	Total
n	2	5	5	4	11	9	14	10	12	72
%	2,8	6,9	6,9	5,6	15,3	12,5	19,4	13,9	16,7	100,0

Summary

	Sum 1-2-3	Sum 4-5-6	Sum 7-8-9
%	16,7	33,3	50,0

Comments
'Strength is unreliable', 'Unfortunately selective muscle strength of only biceps is difficult to test, since brachioradilais muscle can also help flex the elbow.', 'at 1 and 3 months, then - not suitable', 'Biceps recovery will provide a simple proxy for prognostication of upper trunk functions, most important shoulder recovery.', 'but not at later stage.', 'consider assessing biceps MRC-grading at 3 months in babies with complete NBPP', 'I use Gilbert m0-m3', 'impossible to measure MRC strength in an infant.', 'Presence active biceps against gravity.', 'The biceps is examined as a part of the shoulder and elbow functions and the strength per se is not as relevant as the sequential appearance.', 'This is more of an outcome measure', 'We use AMS', 'We would prefer using the term of elbow flexion strength rather than Biceps strenght because it seems impossible to us to distinguish the role of the different possible muscles interventing in elbow flexion (ie: Biceps, Brachialis, Brachio radialis, médial épitrochlean muscles, even lateral condylean muscles)'

Testing biceps strength is suitable to express initial lesion severity.
Please indicate your opinion...

	1	2	3	4	5	6	7	8	9	
fully disagree	○	○	○	○	○	○	○	○	○	fully agree

Please provide your comments.

How to evaluate the severity of the nerve lesion?
(Not necessarily for indication of nerve surgery only)

Elbow flexion (movement)

Absence or presence of elbow flexion was rated by 68% as a suitable measure to express lesion severity. Usually 'against gravity'. Consensus was not reached.

The results of the previous round for elbow flexion were as follows.
Testing elbow flexion is suitable to express severity of the lesion.

rating	1	2	3	4	5	6	7	8	9	Total
n		3	4	2	6	8	16	12	21	72
%		4,2	5,6	2,8	8,3	11,1	22,2	16,7	29,2	100,0

Summary

	Sum 1-2-3	Sum 4-5-6	Sum 7-8-9
%	9,7	22,2	68,1

Elbow flexion should be preferably assessed at

	n	%
1 month of age	3	4,2
3 months of age	23	31,9
6 months of age	5	6,9
9 months of age	2	2,8
serially at these time points	39	54,2
Total	72	100,0

Comments:
'6 weeks and 3 months, preferably with the forearm in supination', 'as contraction is difficult to judge.', 'co-contraction', 'I check the recovery of biceps', 'Measured against gravity. The severity of the lesion is determind by the combined function of: Shoulder, elbow and hand', 'Need to rule out Brachioradialis', 'often by muscles other than biceps','using AMS score', 'We follow monthly'

Testing elbow flexion is suitable to express severity of the lesion.
Please indicate your opinion…

	1	2	3	4	5	6	7	8	9	
fully disagree	○	○	○	○	○	○	○	○	○	fully agree

Elbow flexion should be preferably assessed at

- ○ 1 month of age
- ○ 3 months of age
- ○ 6 months of age
- ○ 9 months of age
- ○ serially at these time points
- ○ never

Please provide your comments.

How to evaluate the severity of the nerve lesion?
(Not necessarily for indication of nerve surgery only)

Time to recovery of elbow flexion
Time to recovery (which month) of elbow flexion against gravity (i.e. MRC3 or better) was suggested by respondents in the first iPluto round.
In the subsequent round 79% rated this item as suitable to express severity of the lesion. **Consensus was reached.**

rating	**1**	**2**	**3**	**4**	**5**	**6**	**7**	**8**	**9**	Total
n	1		3	3	6	2	20	17	20	72
%	1,4		4,2	4,2	8,3	2,8	27,8	23,6	27,8	100,0

How to evaluate the severity of the nerve lesion?
(Not necessarily for indication of nerve surgery only)

Serial investigation.

In the previous round key-movements were scored to be serially investigated at 1-3-6-9 months to globally express the severity of the lesion and the evolution of spontaneous recovery.

On six movements consensus was reached, these movements are judged as necessary to assess for the severity and evolution of spontaneous recovery.

External rotation (in adduction)

	1	2	3	4	5	6	7	8	9	Total	Sum 7-8-9
n	4		5	3	3	2	13	13	28	71	
%	5,6		7,0	4,2	4,2	2,8	18,3	18,3	39,4	100,0	76,1

Abduction

	1	2	3	4	5	6	7	8	9	Total	Sum 7-8-9
n	1		1		3	5	12	15	34	71	
%	1,4		1,4		4,2	7,0	16,9	21,1	47,9	100,0	85,9

Elbow flexion

	1	2	3	4	5	6	7	8	9	Total	Sum 7-8-9
n					1	3	1	9	57	71	
%					1,4	4,2	1,4	12,7	80,3	100,0	94,4

Wrist extension

	1	2	3	4	5	6	7	8	9	Total	Sum 7-8-9
n			1	2	3	5	10	20	30	71	
%			1,4	2,8	4,2	7,0	14,1	28,2	42,3	100,0	84,5

Finger flexion

	1	2	3	4	5	6	7	8	9	Total	Sum 7-8-9
n		2	3		5	3	14	12	32	71	
%		2,8	4,2		7,0	4,2	19,7	16,9	45,1	100,0	81,7

Finger extension

	1	2	3	4	5	6	7	8	9	Total	Sum 7-8-9
n		1		3	4	7	18	15	23	71	
%		1,4		4,2	5,6	9,9	25,4	21,1	32,4	100,0	78,9

Other items did not reach consensus, these items scored as follows:

External rotation (in abduction)

	1	2	3	4	5	6	7	8	9	Total	Sum 7-8-9
n	7	4	5	4	5	6	12	6	22	71	
%	9,9	5,6	7,0	5,6	7,0	8,5	16,9	8,5	31,0	100,0	56,3

Internal rotation

	1	2	3	4	5	6	7	8	9	Total	Sum 7-8-9
n	12	3	8	4	9	11	9	8	7	71	
%	16,9	4,2	11,3	5,6	12,7	15,5	12,7	11,3	9,9	100,0	33,8

Elbow extension

	1	2	3	4	5	6	7	8	9	Total	Sum 7-8-9
n	2	1	2	2	8	8	15	10	23	71	
%	2,8	1,4	2,8	2,8	11,3	11,3	21,1	14,1	32,4	100,0	67,6

Supination

	1	2	3	4	5	6	7	8	9	Total	Sum 7-8-9
n	7	3	6	3	3	12	16	11	10	71	
%	9,9	4,2	8,5	4,2	4,2	16,9	22,5	15,5	14,1	100,0	52,1

Pronation

	1	2	3	4	5	6	7	8	9	Total	Sum 7-8-9
n	9	6	5	5	10	11	12	8	5	71	
%	12,7	8,5	7,0	7,0	14,1	15,5	16,9	11,3	7,0	100,0	35,2

Wrist flexion

	1	2	3	4	5	6	7	8	9	Total	Sum 7-8-9

	1	2	3	4	5	6	7	8	9	Total	Sum 7-8-9
n	4	6	2	4	10	13	14	10	8	71	
%	5,6	8,5	2,8	5,6	14,1	18,3	19,7	14,1	11,3	100,0	45,1

Thumb flexion

	**1**	**2**	**3**	**4**	**5**	**6**	**7**	**8**	**9**	**Total**	**Sum 7-8-9**
n	4	3	4	5	7	3	17	15	13	71	
%	5,6	4,2	5,6	7,0	9,9	4,2	23,9	21,1	18,3	100,0	63,4

Thumb extension

	**1**	**2**	**3**	**4**	**5**	**6**	**7**	**8**	**9**	**Total**	**Sum 7-8-9**
n	4	2		5	4	6	17	16	17	71	
%	5,6	2,8		7,0	5,6	8,5	23,9	22,5	23,9	100,0	70,4

Which key-movements should be serially investigated at 1-3-6-9 months to globally express the severity of the lesion and the evolution of spontaneous recovery. Please rate again.

Remember: the goal if iPluto is to design a minimal dataset (or score-sheet) so that all investigators gather the same data, and report their outcome for the chosen parameters in scientific papers so comparison will be possibile of a certain treatment strategy. Please try to limit the number of movements to increase completeness of data assessment, while keeping essential parameters in the dataset.

Please indicate which active movements should *additionally* *be part of serial investigation.*
Consesus was reached for External Rotation (in adduction), Abduction, Elbow flexion, Wrist Extension, Finger flexion, Finger extension.

	not necessary 1	2	3	4	5	6	7	8	Indispensable 9
External rotation (in abduction)	○	○	○	○	○	○	○	○	○
Internal rotation	○	○	○	○	○	○	○	○	○
Elbow extension	○	○	○	○	○	○	○	○	○
Supination	○	○	○	○	○	○	○	○	○
Pronation	○	○	○	○	○	○	○	○	○
Wrist flexion	○	○	○	○	○	○	○	○	○
Thumb flexion	○	○	○	○	○	○	○	○	○
Thumb extension	○	○	○	○	○	○	○	○	○

How to evaluate the severity of the nerve lesion?
(Not necessarily for indication of nerve surgery only)

Toronto Test score
The Toronto Test Score (aggregate of AMS score of elbow flexion and elbow, wrist, thumb, and finger extension) was scored as follows.

The Toronto Test Score is a suitable instrument to express initial severity of the lesion.

rating	**1**	**2**	**3**	**4**	**5**	**6**	**7**	**8**	**9**	**Total**	**Sum 7-8-9**
n	8	4	6	5	16	6	10	5	11	71	
%	11,3	5,6	8,5	7,0	22,5	8,5	14,1	7,0	15,5	100,0	36,6

The Toronto Test Score should be preferably assessed at

		%
1 month of age	3	4,2
3 months of age	33	46,5
6 months of age	10	14,1
9 months of age	8	11,3
never	17	23,9
Total	71	100,0

Comments:
'AMS very useful, aggregate flawed', 'Because the TTS includes many extension functions it is appropriate for C5-C6-C7 and total lesions, but not for C5-C6 lesions.', 'difficult to use', 'I do not really know the test. I'm not fond of aggregate scores, as you can't distinguish later on which subscores it consists off.', 'I do not use it regularly', 'I don't have been using it in my practice', 'i don't use it', 'In every visit should be evaluated', 'indication for surgery', 'Long run for a short gain.', 'Only separates upper from global', 'This test can only be undertaken at three months of age', 'We currently do not use this score', 'We use elements but not aggregate score'

The Toronto Test Score is a suitable instrument to express initial severity of the lesion.
Please indicate your opinion…

	1	2	3	4	5	6	7	8	9	
fully disagree	○	○	○	○	○	○	○	○	○	fully agree

Please provide your comments.

How to evaluate the severity of the nerve lesion?
(Not necessarily for indication of nerve surgery only)

Cookie Test
The Cookie Test was rated as follows.

The Cookie Test is a suitable instrument to express initial severity of the lesion.

rating	**1**	**2**	**3**	**4**	**5**	**6**	**7**	**8**	**9**	Total	Sum 7-8-9
n	11	5	7	3	8	10	10	9	8	71	
%	15,5	7,0	9,9	4,2	11,3	14,1	14,1	12,7	11,3	100,0	38,0

The Cookie Test should be preferably assessed at

	n	%
3 months of age	6	8,5
6 months of age	18	25,4
9 months of age	34	47,9
never	13	18,3
Total	71	100,0

Comments:
'3-6 months', '9 mon reconstruction too late', 'biceps activity is the key, not elbow fl', 'Can be difficult prior to 9 months', 'For me it)s the way of testing elbowflexion, so yes. But it should be part of the whole exam.', 'I do not agree to do it at 9 months, it should be done at 6 months', 'not an indicator of initial severity', 'should have had surgery', 'The cookietest is advised at 9 months of age, which is relatively late for indication for nerve surgery.', 'The test ca be done with the dummy or the thumb before', 'Used for need for late surgery not severity', 'We do not use it', 'We rely less upon it now', 'We use towel test instead of cookie test'

The Cookie Test is a suitable instrument to express initial severity of the lesion.
Please indicate your opinion...

	1	2	3	4	5	6	7	8	9	
fully disagree	○	○	○	○	○	○	○	○	○	fully agree

The Cookie Test should be preferably assessed at

- ○ 1 month of age
- ○ 3 months of age
- ○ 6 months of age
- ○ 9 months of age
- ○ never

Please provide your comments.

How to evaluate the severity of the nerve lesion? - Ancillary investigations
(Not necessarily for indication of nerve surgery only)

The results from the previous round were as follows:

MRI or CT-myelography is essential to assess the presence of root avulsions.

rating	**1**	**2**	**3**	**4**	**5**	**6**	**7**	**8**	**9**	Total	Sum 7-8-9
n	7	8	6	2	5	6	9	10	18	71	
%	9,9	11,3	8,5	2,8	7,0	8,5	12,7	14,1	25,4	100,0	52,1

The number of root avulsions is an appropriate way to express lesion severity.

rating	**1**	**2**	**3**	**4**	**5**	**6**	**7**	**8**	**9**	Total	Sum 7-8-9
n	3	3	5		9	3	13	15	20	71	
%	4,2	4,2	7,0		12,7	4,2	18,3	21,1	28,2	100,0	67,6

MRI or CT-myelography is essential to assess the presence of root avulsions.
Please indicate your opinion…

	1	2	3	4	5	6	7	8	9	
fully disagree	○	○	○	○	○	○	○	○	○	fully agree

The number of root avulsions is an appropriate way to express lesion severity.
Please indicate your opinion…

	1	2	3	4	5	6	7	8	9	
fully disagree	○	○	○	○	○	○	○	○	○	fully agree

The following questions concern **how to evaluate treatment outcome**.

How to evaluate treatment outcome? - PROM
(Either after surgery or after spontaneous recovery)

Passive ROM was judged by 76% as essential to be included in outcome evaluation, at least for external rotation, abduction and elbow extension.
Consensus was reached for these items.

External rotation (in adduction)

rating	1	2	3	4	5	6	7	8	9	Total	Sum 7-8-9
n					2	2	8	17	41	70	
%					2,9	2,9	11,4	24,3	58,6	100,0	94,3

Abduction

rating	1	2	3	4	5	6	7	8	9	Total	Sum 7-8-9
n	1		2	1	3	4	13	16	30	70	
%	1,4		2,9	1,4	4,3	5,7	18,6	22,9	42,9	100,0	84,3

Elbow extension

rating	1	2	3	4	5	6	7	8	9	Total	Sum 7-8-9
n	1	1		2	5	4	9	23	25	70	
%	1,4	1,4		2,9	7,1	5,7	12,9	32,9	35,7	100,0	81,4

Results for other passive movements were as follows:
External rotation (in abduction)

rating	1	2	3	4	5	6	7	8	9	Total	Sum 7-8-9
n	1	3	3	2	5	6	11	13	26	70	
%	1,4	4,3	4,3	2,9	7,1	8,6	15,7	18,6	37,1	100,0	71,4

Internal rotation

rating	1	2	3	4	5	6	7	8	9	Total	Sum 7-8-9
n	1	4	3	5	5	13	13	13	13	70	
%	1,4	5,7	4,3	7,1	7,1	18,6	18,6	18,6	18,6	100,0	55,7

Elbow flexion

rating	1	2	3	4	5	6	7	8	9	Total	Sum 7-8-9
n	3	2	2	2	10	7	7	13	24	70	
%	4,3	2,9	2,9	2,9	14,3	10,0	10,0	18,6	34,3	100,0	62,9

Supination

rating	1	2	3	4	5	6	7	8	9	Total	Sum 7-8-9
n	2	1	3	3	4	13	15	19	10	70	
%	2,9	1,4	4,3	4,3	5,7	18,6	21,4	27,1	14,3	100,0	62,9

Pronation

rating	1	2	3	4	5	6	7	8	9	Total	Sum 7-8-9
n	3	1	2	3	6	11	12	19	13	70	
%	4,3	1,4	2,9	4,3	8,6	15,7	17,1	27,1	18,6	100,0	62,9

Wrist flexion

rating	1	2	3	4	5	6	7	8	9	Total	Sum 7-8-9
n	5	1	6	2	13	17	14	8	4	70	
%	7,1	1,4	8,6	2,9	18,6	24,3	20,0	11,4	5,7	100,0	37,1

Wrist extension

rating	1	2	3	4	5	6	7	8	9	Total	Sum 7-8-9
n	5		1	2	12	11	14	15	10	70	
%	7,1		1,4	2,9	17,1	15,7	20,0	21,4	14,3	100,0	55,7

Finger flexion

rating	1	2	3	4	5	6	7	8	9	Total	Sum 7-8-9
n	3		2	3	11	13	14	15	9	70	
%	4,3		2,9	4,3	15,7	18,6	20,0	21,4	12,9	100,0	54,3

Finger extension

rating	1	2	3	4	5	6	7	8	9	Total	Sum 7-8-9
n	3	1	1	3	13	10	20	13	6	70	
%	4,3	1,4	1,4	4,3	18,6	14,3	28,6	18,6	8,6	100,0	55,7

Thumb flexion

rating	1	2	3	4	5	6	7	8	9	Total	Sum 7-8-9
n	4	2	6	5	11	14	13	12	3	70	
%	5,7	2,9	8,6	7,1	15,7	20,0	18,6	17,1	4,3	100,0	40,0

Thumb extension

rating	1	2	3	4	5	6	7	8	9	Total	Sum 7-8-9
n	4	1	5	4	11	11	19	10	5	70	
%	5,7	1,4	7,1	5,7	15,7	15,7	27,1	14,3	7,1	100,0	48,6

Please indicate for **which movements** it is necessary to evaluate passive range of motion.

Remember: the goal if iPluto is to design a minimal dataset (or score-sheet) so that all investigators gather the same data, and report their outcome for the chosen parameters in scientific papers so comparison will be possibile of a certain treatment strategy. Please try to limit the number of movements to increase completeness of data assessment, while keeping essential parameters in the dataset.

Consensus was already reached for external rotation, abduction and elbow extension.

	not necessary 1	2	3	4	5	6	7	8	Indispensable 9
External rotation (in abduction)	○	○	○	○	○	○	○	○	○
Internal rotation	○	○	○	○	○	○	○	○	○
Elbow flexion	○	○	○	○	○	○	○	○	○
Supination	○	○	○	○	○	○	○	○	○
Pronation	○	○	○	○	○	○	○	○	○
Wrist flexion	○	○	○	○	○	○	○	○	○
Wrist extension	○	○	○	○	○	○	○	○	○
Finger flexion	○	○	○	○	○	○	○	○	○
Finger extension	○	○	○	○	○	○	○	○	○
Thumb flexion	○	○	○	○	○	○	○	○	○
Thumb extension	○	○	○	○	○	○	○	○	○

Please provide your comments.

How to evaluate treatment outcome? - AROM in degrees
(Either after surgery or after spontaneous recovery)

Active ROM (in degrees) was judged by 94% as essential to be included in outcome evaluation, for external rotation (both in abduction and adduction), abduction, elbow flexion and extension, wrist extension, finger flexion and extension.

External rotation (in abduction)

rating	1	2	3 4	5	6	7	8	9	Total	Sum 7-8-9
n	1	2	1	5	8	4	13	36	70	
%	1,4	2,9	1,4	7,1	11,4	5,7	18,6	51,4	100,0	75,7

External rotation (in adduction)

rating	1	2	3 4	5	6	7	8	9	Total	Sum 7-8-9
n				2	2	4	18	44	70	
%				2,9	2,9	5,7	25,7	62,9	100,0	94,3

Abduction

rating	1	2	3 4	5	6	7	8	9	Total	Sum 7-8-9
n				1	1	2	18	48	70	
%				1,4	1,4	2,9	25,7	68,6	100,0	97,1

Elbow flexion

rating	1	2	3 4	5	6	7	8	9	Total	Sum 7-8-9
n				1	1	1	15	52	70	
%				1,4	1,4	1,4	21,4	74,3	100,0	97,1

Elbow extension

rating	1	2	3 4	5	6	7	8	9	Total	Sum 7-8-9
n				5	5	9	15	36	70	
%				7,1	7,1	12,9	21,4	51,4	100,0	85,7

Wrist extension

rating	1	2	34	5	6	7	8	9	Total	Sum 7-8-9
n	1	1		2	2	11	20	33	70	
%	1,4	1,4		2,9	2,9	15,7	28,6	47,1	100,0	91,4

Finger flexion

rating	1	2	34	5	6	7	8	9	Total	Sum 7-8-9
n		1	1	3	8	8	18	31	70	
%		1,4	1,4	4,3	11,4	11,4	25,7	44,3	100,0	81,4

Finger extension

rating	1	2	34	5	6	7	8	9	Total	Sum 7-8-9
n			1	3	6	11	23	26	70	
%			1,4	4,3	8,6	15,7	32,9	37,1	100,0	85,7

Results for other active movements were as follows:

Internal rotation

rating	1	2	3	4	5	6	7	8	9	Total	Sum 7-8-9
n		1	3	1	5	15	9	12	24	70	
%		1,4	4,3	1,4	7,1	21,4	12,9	17,1	34,3	100,0	64,3

Supination

rating	1	2	3	4	5	6	7	8	9	Total	Sum 7-8-9
n		1	1	2	4	11	13	11	27	70	
%		1,4	1,4	2,9	5,7	15,7	18,6	15,7	38,6	100,0	72,9

Pronation

rating	1	2	3	4	5	6	7	8	9	Total	Sum 7-8-9
n	1	1	2	2	6	11	14	11	22	70	
%	1,4	1,4	2,9	2,9	8,6	15,7	20,0	15,7	31,4	100,0	67,1

Wrist flexion

rating	1	2	3	4	5	6	7	8	9	Total	Sum 7-8-9
n	2	2	1	1	7	12	11	15	19	70	
%	2,9	2,9	1,4	1,4	10,0	17,1	15,7	21,4	27,1	100,0	64,3

Thumb flexion

rating	1	2	3	4	5	6	7	8	9	Total	Sum 7-8-9
n	1	2	2		5	11	8	17	24	70	
%	1,4	2,9	2,9		7,1	15,7	11,4	24,3	34,3	100,0	70,0

Thumb extension

rating	1	2	3	4	5	6	7	8	9	Total	Sum 7-8-9
n	1	1	1	1	5	9	8	19	25	70	
%	1,4	1,4	1,4	1,4	7,1	12,9	11,4	27,1	35,7	100,0	74,3

Please indicate for **which movements** it is necessary to evaluate active range of motion (in degrees) as treatment outcome.

Remember: the goal if iPluto is to design a minimal dataset (or score-sheet) so that all investigators gather the same data, and report their outcome for the chosen parameters in scientific papers so comparison will be possibile of a certain treatment strategy. Please try to limit the number of movements to increase completeness of data assessment, while keeping essential parameters in the dataset.

Consensus was reached to include external rotation (both in abduction and adduction), abduction, elbow flexion and extension, wrist extension, finger flexion and extension.

	not necessary 1	2	3	4	5	6	7	8	Indispensable 9
Internal rotation	○	○	○	○	○	○	○	○	○
Supination	○	○	○	○	○	○	○	○	○
Pronation	○	○	○	○	○	○	○	○	○
Wrist flexion	○	○	○	○	○	○	○	○	○
Thumb flexion	○	○	○	○	○	○	○	○	○
Thumb extension	○	○	○	○	○	○	○	○	○

Please provide your comments.

How to evaluate treatment outcome? - AROM in AMS
(Either after surgery or after spontaneous recovery)

Active range of motion measured according to the Active Movement System (which originated in Toronto) was scored in the previous round as follows.

Summary of the AMS: see here

Active ROM (expressed according to the Active Movement System AMS) was judged as follows. Only abduction reached consensus (76%) as essential to be included in outcome evaluation.

Active range of motion (expressed as AMS) is an appropriate measure to express treatment outcome.

	1	2	3	4	5	6	7	8	9	Total	Sum 7-8-9
n	4	2	3	4	11	8	11	5	22	70	
%	5,7	2,9	4,3	5,7	15,7	11,4	15,7	7,1	31,4	100,0	54,3

Active range of motion (expressed as AMS) is essential to be included in a minimal dataset to publish or compare results.

n	1	2	3	4	5	6	7	8	9	Total	Sum 7-8-9
%	8	3	6	3	12	4	5	8	21	70	
	11,4	4,3	8,6	4,3	17,1	5,7	7,1	11,4	30,0	100,0	48,6

Comments
'Ambivalent, limited by PROM, widely used', 'AMS is optional', 'good for upper lesion', 'I have not been using this System No Valid answer', 'If thorough examination with AROM and PROM we don´t think AMS is needed. I may be of importance for the youngest children', 'The AMS is not enough sensible, and there is a problem with the 5 – 6- 7 cotation in comparison with the other side when the child grows between 0 and 2 years old.', 'The AMS may be a good system to express motion in younger children, but not as 'final' outcome measure. Correlation with functional state only applies for higher scores.', 'Total score requires all movements.', 'We generally do not use AMS', 'We have modified AMS to include degrees', 'when taken into account the presence of contractures.'

The individual movements were rated as follows

External rotation (in abduction)

rating	1	2	3	4	5	6	7	8	9	Total	Sum 7-8-9
n	8	3	5	2	5	8	6	11	22	70	
%	11,4	4,3	7,1	2,9	7,1	11,4	8,6	15,7	31,4	100,0	55,7

External rotation (in adduction)

rating	1	2	3	4	5	6	7	8	9	Total	Sum 7-8-9
n	7		2	1	7	2	7	13	31	70	
%	10,0		2,9	1,4	10,0	2,9	10,0	18,6	44,3	100,0	72,9

Abduction

rating	1	2	3	4	5	6	7	8	9	Total	Sum 7-8-9
n	7		1	1	5	3	6	14	33	70	
%	10,0		1,4	1,4	7,1	4,3	8,6	20,0	47,1	100,0	75,7

Internal rotation

rating	1	2	3	4	5	6	7	8	9	Total	Sum 7-8-9
n	7		4	3	10	7	10	10	19	70	
%	10,0		5,7	4,3	14,3	10,0	14,3	14,3	27,1	100,0	55,7

Elbow flexion

rating	1	2	3	4	5	6	7	8	9	Total	Sum 7-8-9
n	8		1	1	6	4		12	38	70	
%	11,4		1,4	1,4	8,6	5,7		17,1	54,3	100,0	71,4

Elbow extension

rating	1	2	3	4	5	6	7	8	9	Total	Sum 7-8-9
n	8		3	1	6	5	8	9	30	70	
%	11,4		4,3	1,4	8,6	7,1	11,4	12,9	42,9	100,0	67,1

Supination

rating	1	2	3	4	5	6	7	8	9	Total	Sum 7-8-9
n	8	1	4	5	9	6	7	15	15	70	
%	11,4	1,4	5,7	7,1	12,9	8,6	10,0	21,4	21,4	100,0	52,9

Pronation

rating	1	2	3	4	5	6	7	8	9	Total	Sum 7-8-9
n	9	1	4	3	10	9	7	12	15	70	
%	12,9	1,4	5,7	4,3	14,3	12,9	10,0	17,1	21,4	100,0	48,6

Wrist flexion

rating	1	2	3	4	5	6	7	8	9	Total	Sum 7-8-9
n	9		5	2	11	7	10	11	15	70	
%	12,9		7,1	2,9	15,7	10,0	14,3	15,7	21,4	100,0	51,4

Wrist extension

rating	1	2	3	4	5	6	7	8	9	Total	Sum 7-8-9
n	9		3	1	8	3	6	14	26	70	
%	12,9		4,3	1,4	11,4	4,3	8,6	20,0	37,1	100,0	65,7

Finger flexion

rating	1	2	3	4	5	6	7	8	9	Total	Sum 7-8-9
n	8		3	1	9	4	6	12	27	70	
%	11,4		4,3	1,4	12,9	5,7	8,6	17,1	38,6	100,0	64,3

Finger extension

rating	1	2	3	4	5	6	7	8	9	Total	Sum 7-8-9
n	9		3	2	7	4	7	16	22	70	
%	12,9		4,3	2,9	10,0	5,7	10,0	22,9	31,4	100,0	64,3

Thumb flexion

rating	1	2	3	4	5	6	7	8	9	Total	Sum 7-8-9
n	9	1	4	1	12	5	6	13	19	70	
%	12,9	1,4	5,7	1,4	17,1	7,1	8,6	18,6	27,1	100,0	54,3

Thumb extension

rating	1	2	3	4	5	6	7	8	9	Total	Sum 7-8-9
n	9		3	2	11	5	7	11	22	70	
%	12,9		4,3	2,9	15,7	7,1	10,0	15,7	31,4	100,0	57,1

Active range of motion (expressed as AMS) is an appropriate measure to express treatment outcome.
Please indicate your opinion...

	1	2	3	4	5	6	7	8	9	
fully disagree	○	○	○	○	○	○	○	○	○	fully agree

Active range of motion (expressed as AMS) is essential to be included in a minimal dataset to publish or compare results.
Please indicate your opinion...

	1	2	3	4	5	6	7	8	9	
fully disagree	○	○	○	○	○	○	○	○	○	fully agree

Please indicate for **which movements** it is necessary to evaluate active range of motion (in AMS).

	not necessary 1	2	3	4	5	6	7	8	Indispensable 9
External rotation (in abduction)	○	○	○	○	○	○	○	○	○
External rotation (in adduction)	○	○	○	○	○	○	○	○	○
Abduction	○	○	○	○	○	○	○	○	○
Internal rotation	○	○	○	○	○	○	○	○	○
Elbow flexion	○	○	○	○	○	○	○	○	○
Elbow extension	○	○	○	○	○	○	○	○	○
Supination	○	○	○	○	○	○	○	○	○
Pronation	○	○	○	○	○	○	○	○	○
Wrist flexion	○	○	○	○	○	○	○	○	○
Wrist extension	○	○	○	○	○	○	○	○	○
Finger flexion	○	○	○	○	○	○	○	○	○
Finger extension	○	○	○	○	○	○	○	○	○
Thumb flexion	○	○	○	○	○	○	○	○	○
Thumb extension	○	○	○	○	○	○	○	○	○

Please provide your comments.

How to evaluate treatment outcome? - Muscle Force
(Either after surgery or after spontaneous recovery)

Muscle force (MRC-grading) was scored as follows.

Muscle force (MRC grading) was judged as follows. Only evaluation of biceps strength reached consensus (76%) as essential to be included in outcome evaluation.

Muscle force (MRC) is an appropriate measure to express treatment outcome.

rating	1	2	3	4	5	6	7	8	9	TotalSum	7-8-9
n	5	4	5	1	5	6	12	16	16	70	
%	7,1	5,7	7,1	1,4	7,1	8,6	17,1	22,9	22,9	100,0	62,9

Muscle force (MRC) is essential to be included in a minimal dataset to publish or compare results.

rating	1	2	3	4	5	6	7	8	9	TotalSum	7-8-9
n	7	4	8	2	7	2	10	13	17	70	
%	10,0	5,7	11,4	2,9	10,0	2,9	14,3	18,6	24,3	100,0	57,1

For the individual muscles, scores were as follows

Deltoid muscle

rating	1	2	3	4	5	6	7	8	9	TotalSum	7-8-9
n	6	2	6	4	3	4	12	16	17	70	
%	8,6	2,9	8,6	5,7	4,3	5,7	17,1	22,9	24,3	100,0	64,3

Biceps muscle

rating	1	2	3	4	5	6	7	8	9	TotalSum	7-8-9
n	5	1	4	4	1	2	6	13	34	70	
%	7,1	1,4	5,7	5,7	1,4	2,9	8,6	18,6	48,6	100,0	75,7

Triceps muscle

rating	1	2	3	4	5	6	7	8	9	TotalSum	7-8-9
n	5	3	5	6	6	4	13	15	13	70	
%	7,1	4,3	7,1	8,6	8,6	5,7	18,6	21,4	18,6	100,0	58,6

Wrist extensors

rating	1	2	3	4	5	6	7	8	9	TotalSum	7-8-9
n	7	2	3	3	4	4	12	14	21	70	
%	10,0	2,9	4,3	4,3	5,7	5,7	17,1	20,0	30,0	100,0	67,1

Grip strength

rating	1	2	3	4	5	6	7	8	9	TotalSum	7-8-9
n	5	2	7	4	3	2	9	14	24	70	
%	7,1	2,9	10,0	5,7	4,3	2,9	12,9	20,0	34,3	100,0	67,1

Comments:
'Age dependent', 'Although important, diffucult to measure', 'Best after 3 years', 'Depend of type of injury if total or partial', 'Difficult to apply 0-5 scale in children', 'difficult to evaluate', 'finger/wrist flexors: 8', 'Hard to obtain until older', 'MRC not reliable with young children', 'older children (adult BMRC). AMS young', 'The deltoid alone is hard to examine in small children. Maybe it is better to test the strenght in shoulder abduction/flexion movment instead', 'The MRC system might not discriminate very good, but it is useful to correlate with functional outcomes.', 'There is difficulties of sensibility between 4 and 5, and inter asseser. The grip strengh must be évaluate with dynamometer, and thumb flexion with pinch strengh.'

Muscle force (MRC) is an appropriate measure to express treatment outcome.
Please indicate your opinion...

	1	2	3	4	5	6	7	8	9	
fully disagree	○	○	○	○	○	○	○	○	○	fully agree

Muscle force (MRC) is essential to be included in a minimal dataset to publish or compare results.
Please indicate your opinion...

	1	2	3	4	5	6	7	8	9	
fully disagree	○	○	○	○	○	○	○	○	○	fully agree

Please indicate for **which muscles** it is necessary to evaluate **muscle force**.

Remember: for biceps musle consensus was reached in the previous round.

not necessary 1	2	3	4	5	6	7	8	Indispensable 9

Deltoid muscle	○	○	○	○	○	○	○	○	○
Triceps muscle	○	○	○	○	○	○	○	○	○
Wrist extensors	○	○	○	○	○	○	○	○	○
Grip strength	○	○	○	○	○	○	○	○	○

Please provide your comments.

How to evaluate treatment outcome? - Scoring systems
(Either after surgery or after spontaneous recovery)

The Mallet Score was judged by 76% as an essential outcome measure, and there was agreement that all individual subscores should be included.
There was nog agreement on the modified Mallet score, which adds hand-to-belly.
The majority did not appreciate the aggregate / sum score.

The Mallet Score is an appropriate measure to express treatment outcome

rating	1	2	3	4	5	6	7	8	9	Total	Sum 7-8-9
n			1	2	8	2	13	22	22	70	
%			1,4	2,9	11,4	2,9	18,6	31,4	31,4	100,0	81,4

The Mallet Score is essential to be included in a minimal dataset to publish or compare results.

rating	1	2	3	4	5	6	7	8	9	Total	Sum 7-8-9
n	1	1	1	3	6	5	8	22	23	70	
%	1,4	1,4	1,4	4,3	8,6	7,1	11,4	31,4	32,9	100,0	75,7

Please indicate which way you prefer to use the Mallet-score - Each of 5 subscores

rating	1	2	3	4	5	6	7	8	9	Total	Sum 7-8-9
n	3	2	3	1	6	2	9	20	24	70	
%	4,3	2,9	4,3	1,4	8,6	2,9	12,9	28,6	34,3	100,0	75,7

Please indicate which way you prefer to use the Mallet-score - Add item: hand to belly

rating	1	2	3	4	5	6	7	8	9	Total	Sum 7-8-9
n	5	3	3	2	6	4	7	17	23	70	
%	7,1	4,3	4,3	2,9	8,6	5,7	10,0	24,3	32,9	100,0	67,1

Please indicate which way you prefer to use the Mallet-score - Aggregate (sum score)

rating	1	2	3	4	5	6	7	8	9	Total	Sum 7-8-9
n	12	5	4	2	8	7	3	19	10	70	
%	17,1	7,1	5,7	2,9	11,4	10,0	4,3	27,1	14,3	100,0	45,7

Comments:
'Good for shoulder, measures what is need', 'I score it always on 20 never use 25', 'It could be better, but everybody knows it', 'Mallet Score only in older kids', 'Mallet/score does not tell me anything about handfunction, so it is not an adequate outcome measure', 'only for upper OBPL (C5/6/7 Lesions)', 'scores 0, 1 and 5 are almost never used', 'The Mallet system is very simple, and easy to apply. In a sum score one looses the different movements.', 'The most important in Mallet score is regarding to us: Trumpet sign and hand to neck. These moves are not covered by AROM.', 'We don't use this routinely', 'we use 5 subscores 1-3 out of 15!'

Please indicate **which way** you prefer to use the **Mallet-score,** additional to the individual subscores, for which consensus was reached.

	not necessary 1	2	3	4	5	6	7	8	Indispensable 9
Add item: hand to belly (Modified Mallet)	○	○	○	○	○	○	○	○	○
Aggregate (sum score)	○	○	○	○	○	○	○	○	○

Please provide your comments.

How to evaluate treatment outcome? - Scoring systems
(Either after surgery or after spontaneous recovery)

The Gilbert Elbow Score was scored as follows in the previous round, many do not consider it valuable (scores 7-8-9), but as many participants scored neutral, consensus was not reached.

The Gilbert Elbow Score is an appropriate measure to express treatment outcome

rating	1	2	3	4	5	6	7	8	9	Total	Sum 7-8-9
n	13	8	10	9	7	10	5	4	4	70	
%	18,6	11,4	14,3	12,9	10,0	14,3	7,1	5,7	5,7	100,0	18,6

The Gilbert Elbow Score is essential to be included in a minimal dataset to publish or compare results.

rating	1	2	3	4	5	6	7	8	9	Total	Sum 7-8-9
n	16	11	10	8	10	4	5	2	4	70	
%	22,9	15,7	14,3	11,4	14,3	5,7	7,1	2,9	5,7	100,0	15,7

Comments:
'A sum score of flexion and extension does not discriminate where the functional problem exists.', 'i don't know it', 'I don't know it-use it.', 'Prefer P/AROM', 'Questionable usefulness', 'Score good when function poor.', 'unfamiliar with this test', 'We do not use this scale routinely'

The Gilbert Elbow Score is an appropriate measure to express treatment outcome.
Please indicate your opinion...

	1	2	3	4	5	6	7	8	9	
fully disagree	○	○	○	○	○	○	○	○	○	fully agree

The Gilbert Elbow Score is essential to be included in a minimal dataset to publish or compare results.
Please indicate your opinion...

	1	2	3	4	5	6	7	8	9	
fully disagree	○	○	○	○	○	○	○	○	○	fully agree

Please provide your comments.

How to evaluate treatment outcome? - Scoring systems
(Either after surgery or after spontaneous recovery)

The Raimondi Hand Score was scored as follows in the previous round:

The Raimondi Hand Score is an appropriate measure to express treatment outcome.

rating	1	2	3	4	5	6	7	8	9	Total	Sum 7-8-9
n	9	7	8	5	13	3	10	8	7	70	
%	12,9	10,0	11,4	7,1	18,6	4,3	14,3	11,4	10,0	100,0	35,7

The Raimondi Hand Score is essential to be included in a minimal dataset to publish or compare results.

rating	1	2	3	4	5	6	7	8	9	Total	Sum 7-8-9
n	12	7	11	3	14	2	7	8	6	70	
%	17,1	10,0	15,7	4,3	20,0	2,9	10,0	11,4	8,6	100,0	30,0

Comments:
'Although the Raimondi score lacks finesse, it is the only available simple score to express hand function.', 'if evaluating hand', 'More accurate than strength for outcome', 'Not essential in my opinion', 'Of course, only when lower roots are affected', 'specific for hand', 'We do not use this scale routinely'

The Raimondi Hand Score is an appropriate measure to express treatment outcome.
Please indicate your opinion...

	1	2	3	4	5	6	7	8	9	
fully disagree	○	○	○	○	○	○	○	○	○	fully agree

The Raimondi Hand Score is essential to be included in a minimal dataset to publish or compare results.
Please indicate your opinion...

	1	2	3	4	5	6	7	8	9	
fully disagree	○	○	○	○	○	○	○	○	○	fully agree

Please provide your comments.

How to evaluate treatment outcome? - Scoring systems
(Either after surgery or after spontaneous recovery)

The **Brachial Plexus Outcome Measure (BPOM)** was scored as follows in the previous round:

The BPOM is an appropriate measure to express treatment outcome.

rating	1	2	3	4	5	6	7	8	9	Total	Sum 7-8-9
n	13	6	8	2	16	6	4	6	9	70	
%	18,6	8,6	11,4	2,9	22,9	8,6	5,7	8,6	12,9	100,0	27,1

The BPOM is essential to be included in a minimal dataset to publish or compare results.

rating	1	2	3	4	5	6	7	8	9	Total	Sum 7-8-9
n	16	8	8	5	12	2	8	6	5	70	
%	22,9	11,4	11,4	7,1	17,1	2,9	11,4	8,6	7,1	100,0	27,1

Comments:
'18 years may be better age to assess - i.e. after skeletal maturity.', 'Adds unnecessary layer', 'AHA is most important for Children with total injuries or C5-7 lesions', 'Although in setup it seems an appropriate outcome measure, many of the items evaluate the assisting hand as it where the dominant hand (e.g. control of a computer mouse).', 'as early as particular tasks are possibl', 'I do not know well', 'i don't know it', 'i don't use BPOM', 'i don't use it', 'I don't know this measure', 'i dont know it', 'Requires added time/resources in clinic', 'We do not use', 'We don´t have enough experience of this scoring system to answer the question'

The BPOM is an appropriate measure to express treatment outcome.
Please indicate your opinion...

	1	2	3	4	5	6	7	8	9	
fully disagree	○	○	○	○	○	○	○	○	○	fully agree

The BPOM is essential to be included in a minimal dataset to publish or compare results.
Please indicate your opinion...

	1	2	3	4	5	6	7	8	9	
fully disagree	○	○	○	○	○	○	○	○	○	fully agree

The BPOM should be preferably assessed at the age of (multiple answers possible)

- ☐ 1 year
- ☐ 2 years
- ☐ 3 years
- ☐ 5 years
- ☐ 7 years
- ☐ 15 years
- ☐ never

Please provide your comments.

How to evaluate treatment outcome? - Scoring systems
(Either after surgery or after spontaneous recovery)

The **Assisting Hand Assessment (AHA)** was scored as follows in the previous round:

The AHA is an appropriate measure to express treatment outcome.

rating	1	2	3	4	5	6	7	8	9	TotalSum	7-8-9
n	10	6	11	4	11	9	8	7	4	70	
%	14,3	8,6	15,7	5,7	15,7	12,9	11,4	10,0	5,7	100,0	27,1

The AHA is essential to be included in a minimal dataset to publish or compare results.

rating	1	2	3	4	5	6	7	8	9	TotalSum	7-8-9
n	13	7	13	7	9	5	5	8	3	70	
%	18,6	10,0	18,6	10,0	12,9	7,1	7,1	11,4	4,3	100,0	22,9

Comments:
'depends on project as to if it is needed', 'I don't know it.', 'I don't use AHA', 'i don't use it', 'In it´s current form AHA is only working for total injuries. Not realistic to use in a minimal dataset.', 'most important for children with total injuries or C5-7 lesions', 'Most of recovery, even after surgery is completed. At this moment it is usefull to see how much and how affective the involved hand is being used.', 'not aware', 'Not easy to do, and need more time', 'Not familiar yet with this scoring systems', 'Require huge OP/PT backup', 'The AHA is a fantastic score, but it is not very applicable in routine clinical practice as it is too long to complete.', 'Time/resource intensive, only for research', 'We do not use'

The AHA is an appropriate measure to express treatment outcome.
Please indicate your opinion…

	1	2	3	4	5	6	7	8	9	
fully disagree	○	○	○	○	○	○	○	○	○	fully agree

The AHA is essential to be included in a minimal dataset to publish or compare results.
Please indicate your opinion…

	1	2	3	4	5	6	7	8	9	
fully disagree	○	○	○	○	○	○	○	○	○	fully agree

The AHA should be preferably assessed at the age of (multiple answers possible)

- ☐ 1 year
- ☐ 2 years
- ☐ 3 years
- ☐ 5 years
- ☐ 7 years
- ☐ 15 years
- ☐ never

Please provide your comments.

How to evaluate treatment outcome? - Scoring systems
(Either after surgery or after spontaneous recovery)

The **Nine hole peg test** was scored as follows in the previous round:

The Nine hole peg test is an appropriate measure to express treatment outcome.

rating	1	2	3	4	5	6	7	8	9	Total	Sum 1-2-3
n	18	14	16	5	9	3	1	3	1	70	
%	25,7	20,0	22,9	7,1	12,9	4,3	1,4	4,3	1,4	100,0	68,6

The Nine hole peg test is essential to be included in a minimal dataset to publish or compare results.

rating	1	2	3	4	5	6	7	8	9	Total	Sum 1-2-3
n	24	13	17	3	6	3	1	3		70	
%	34,3	18,6	24,3	4,3	8,6	4,3	1,4	4,3		100,0	77,1

Comments:
'Don't increase complexity and exclude', 'i don't use it', 'i don't use nine hole', 'No experience of this test', 'not at all useful for this group', 'Not familiar with this system', 'To difficult to do for young child. The box and block at the age of 5 or the perdue pegboard at the age of 7 are better.', 'to use to assess depends on study', 'We do not use', 'We do not use this'

There was consensus as 77% rated this test as not to be included in a minimal dataset.

How to evaluate treatment outcome? - Sensation
(Either after surgery or after spontaneous recovery)

Semmes Weinstein filaments (SWf) to evaluate senasation was scored as follows in the previous round:

SWf is an appropriate measure to express treatment outcome.

rating	1	2	3	4	5	6	7	8	9	Total	Sum 7-8-9
n	11	6	13	4	11	7	12	3	3	70	
%	15,7	8,6	18,6	5,7	15,7	10,0	17,1	4,3	4,3	100,0	25,7

SWf is essential to be included in a minimal dataset to publish or compare results.

rating	1	2	3	4	5	6	7	8	9	Total	Sum 7-8-9
n	14	14	11	1	11	5	9	3	2	70	
%	20,0	20,0	15,7	1,4	15,7	7,1	12,9	4,3	2,9	100,0	20,0

Comments:
'Before five years, we can use evaluation of hot and warm or trying to know with game.', 'Before is difficult', 'Completely age dependent', 'difficult for young children', 'i don't use SWf', 'It is the only validated measure for sensation.', 'No under 3 per literature', 'Not a suitable test for small children and by the time the child can cooperate so then there is nothing to compare before or after intervention..', 'not possible under 5 to 7 years', 'Not valid before 5-7y of age', 'Sensation rarely a problem.', 'We do not use', 'We don´t use this for children'

SWf is an appropriate measure to express treatment outcome.
Please indicate your opinion…

	1	2	3	4	5	6	7	8	9	
fully disagree	○	○	○	○	○	○	○	○	○	fully agree

SWf is essential to be included in a minimal dataset to publish or compare results.
Please indicate your opinion…

	1	2	3	4	5	6	7	8	9	
fully disagree	○	○	○	○	○	○	○	○	○	fully agree

SWfilaments should be preferably assessed at the age of (multiple answers possible)

- ☐ 1 year
- ☐ 2 years
- ☐ 3 years
- ☐ 5 years
- ☐ 7 years
- ☐ 15 years
- ☐ never

Please provide your comments.

How to evaluate treatment outcome? - Sensation
(Either after surgery or after spontaneous recovery)

2 point discrimination (2PD) for evaluation of sensation was scored as follows in the previous round:

2PD is an appropriate measure to express treatment outcome.

rating	1	2	3	4	5	6	7	8	9	Total	Sum 7-8-9
n	11	6	11	7	11	7	10	4	3	70	
%	15,7	8,6	15,7	10,0	15,7	10,0	14,3	5,7	4,3	100,0	24,3

2PD is essential to be included in a minimal dataset to publish or compare results.

rating	1	2	3	4	5	6	7	8	9	Total	Sum 7-8-9
n	13	7	15	6	7	6	9	5	2	70	
%	18,6	10,0	21,4	8,6	10,0	8,6	12,9	7,1	2,9	100,0	22,9

Comments:
'at age > 5years', 'It is a validated measure for sensation.', 'not possible under 5-7 years', 'Not valid before 5-7y of age', 'Only if sensory-related issues', 'only older kids', 'Sensation rarely a problem.', 'We do not use'

2PD is an appropriate measure to express treatment outcome.
Please indicate your opinion…

	1	2	3	4	5	6	7	8	9	
fully disagree	○	○	○	○	○	○	○	○	○	fully agree

2PD is essential to be included in a minimal dataset to publish or compare results.
Please indicate your opinion…

	1	2	3	4	5	6	7	8	9	
fully disagree	○	○	○	○	○	○	○	○	○	fully agree

2PD should be preferably assessed at the age of (multiple answers possible)

- ☐ 1 year
- ☐ 2 years
- ☐ 3 years
- ☐ 5 years
- ☐ 7 years
- ☐ 15 years
- ☐ never

Please provide your comments.

How to evaluate treatment outcome? - Pain
(Either after surgery or after spontaneous recovery)

Pain questionnaires were scored as follows in the previous round:

Pain questionnaires is an appropriate measure to express treatment outcome.

rating	**1**	**2**	**3**	**4**	**5**	**6**	**7**	**8**	**9**	TotalSum	7-8-9
n	7	5	8	4	9	5	14	9	9	70	
%	10,0	7,1	11,4	5,7	12,9	7,1	20,0	12,9	12,9	100,0	45,7

Pain questionnaires is essential to be included in a minimal dataset to publish or compare results.

rating	**1**	**2**	**3**	**4**	**5**	**6**	**7**	**8**	**9**	TotalSum	7-8-9
n	12	4	9	6	8	5	11	9	6	70	
%	17,1	5,7	12,9	8,6	11,4	7,1	15,7	12,9	8,6	100,0	37,1

Comments:
'Before 3 years, the parents respond. After, it's better to work with them', 'IF longer follow up test at 18', 'rarely encounter in children', 'Two kinds of pain exist: neuralgic pain and surmenage. The latter is a major problem in the older children.', 'VAS scale would be enough', 'very infrequent that there is pain', 'we use PODCI which has pain questions'

Pain questionnaires is an appropriate measure to express treatment outcome.
Please indicate your opinion...

	1	2	3	4	5	6	7	8	9	
fully disagree	○	○	○	○	○	○	○	○	○	fully agree

Pain questionnaires is essential to be included in a minimal dataset to publish or compare results.
Please indicate your opinion...

	1	2	3	4	5	6	7	8	9	
fully disagree	○	○	○	○	○	○	○	○	○	fully agree

Pain questionnaires should be preferably assessed at the age of (multiple answers possible)

- ☐ 1 year
- ☐ 2 years
- ☐ 3 years
- ☐ 5 years
- ☐ 7 years
- ☐ 15 years
- ☐ never

Please provide your comments.

How to evaluate treatment outcome? - PROMs
(Either after surgery or after spontaneous recovery)

PROMs (Patient Reported Outcome Measures) was scored as follows in the previous round:

Assessment of PROMs (Patient Reported Outcome Measures) is an appropriate method to express treatment outcome.

rating	1	2	3	4	5	6	7	8	9	Total	Sum 7-8-9
n	5	5	7	6	8	8	12	10	9	70	
%	7,1	7,1	10,0	8,6	11,4	11,4	17,1	14,3	12,9	100,0	44,3

PROMs (Patient Reported Outcome Measures) are essential to be included in a minimal dataset to publish or compare results.

rating	1	2	3	4	5	6	7	8	9	Total	Sum 7-8-9
n	6	7	8	3	14	4	10	9	9	70	
%	8,6	10,0	11,4	4,3	20,0	5,7	14,3	12,9	12,9	100,0	40,0

I have / Our center has sufficient experience with different PROMs to judge which PROMS are the most appropriate.

rating	1	2	3	4	5	6	7	8	9	Total	Sum 7-8-9
n	21	10	13	3	8	3	3	4	5	70	
%	30,0	14,3	18,6	4,3	11,4	4,3	4,3	5,7	7,1	100,0	17,1

It is striking that only 17% judged themselves as capable to judge the validity of different PROMs, although between 40 and 50% of you judged PROMs to be important.
We decided to exclude PROMs from the general iPluto questionnaires to build a minimal dataset.

We feel, however, that PROMs may be an appropriate way to measure outcome as part of an *extended* dataset. As many different scoring lists are available, we seek the help of those of you who are interested in PROMs to be part of a spin-off project on the topic of PROMs.

Concerning the scoring of PROMs

- ○ I am not available / capable for the evaluation of PROMs
- ○ I am willing to contribute to the evaluation of PROMs (which implies to invest time to study unknown PROMs)
- ○ I refer to the person in my center who has the best knowledge to contribute to this topic:(name + email-address)

The iPluto age proposal for timing of evaluation

Standardized time points for the collection of data should be used to compare results. iPluto proposes to use the age of the infant, and not the follow-up time after a specific intervention. Our first proposal in the first round was to evaluate at the age of 1 / 3 / 5 / 7 years.

- one year, because this is a plateau for spontaneous neurological recovery. Additionally, this time point may serve as a baseline before results of treatment interventions will have taken an effect.
- three years, because this reflects the final stage of spontaneous recovery, and a plateau for shoulder function in children who were treated with early nerve reconstruction.
- five years, because by this time an end-stage is reached for nerve reconstruction of the shoulder, and a plateau is reached for hand function. Additionally, it could serve as a pre-school assessment of function.
- seven years, because by this time most secondary surgical procedures will have been performed and an end stage for hand function is reached. Limitations in the first year(s) of school and during leisure (e.g. sports) can be identified at this age because of sufficient cooperation.

In the first round 63/68 (93%) supported this concept. Many participants suggested to add a time point at 2 years of age, and one as teenager, e.g. at 15 years of age.
Please note that time points before the age of one year will be collected to serve as baseline to express lesion severity.

There was consensus on the standardized time-points of 1-3-5-7 years to evaluate outcome. There was a majority in favor to add 2 years and 15 years, but not reaching 75% consensus.

1 year												
	rating	1	2	3	4	5	6	7	8	9	TotalSum	7-8-9
	n	6	1			4	3	6	9	41	70	
	%	8,6	1,4			5,7	4,3	8,6	12,9	58,6	100,0	80,0
2 years												
	rating	1	2	3	4	5	6	7	8	9	TotalSum	7-8-9
	n	7	4	3	2	5	1	10	9	29	70	
	%	10,0	5,7	4,3	2,9	7,1	1,4	14,3	12,9	41,4	100,0	68,6
3 years												
	rating	1	2	3	4	5	6	7	8	9	TotalSum	7-8-9
	n	3	1		1	4	2	7	8	44	70	
	%	4,3	1,4		1,4	5,7	2,9	10,0	11,4	62,9	100,0	84,3
5 years												
	rating	1	2	3	4	5	6	7	8	9	TotalSum	7-8-9
	n	2	1			2	2	9	7	47	70	
	%	2,9	1,4			2,9	2,9	12,9	10,0	67,1	100,0	90,0
7 years												
	rating	1	2	3	4	5	6	7	8	9	TotalSum	7-8-9
	n	4			1	4	5	8	8	40	70	
	%	5,7			1,4	5,7	7,1	11,4	11,4	57,1	100,0	80,0
15 years												
	rating	1	2	3	4	5	6	7	8	9	TotalSum	7-8-9
	n	4	1			5	8	6	9	37	70	
	%	5,7	1,4			7,1	11,4	8,6	12,9	52,9	100,0	74,3

Please indicate at **which age** it is desired to evaluate outcome.
There was already consensus on 1-3-5-7 years.

	not necessary 1	2	3	4	5	6	7	8	Indispensable 9
2 year	○	○	○	○	○	○	○	○	○
15 year	○	○	○	○	○	○	○	○	○

Please provide your comments.

How to score muscles / movements / motion ?

The evaluation of active recovery of muscles / movements is done and scored differently.

Three main systems are currently used:

- active movement in degrees
- Active Movement System (from Toronto)
- muscle force (MRC-score)

aROM in degrees scored high, but there was no consensus in the first round for AMS or MRC.

Al Qattan tried to combine both in one hybrid system, but this has not been adopted widely.
(Al-Qattan MM. Assessment of the motor power in older children with obstetric brachial plexus palsy. J Hand Surg Br. 2003 Feb;28(1):46-9.)

It would look something like this in (amended):

	**Medical Research Council system**		**Hybrid system**		**Active Movement System**
0	no contraction or flicker of contraction	0		0	no contraction
1	contraction without movement	1		1	contraction, no motion
2	active movement with gravity eliminated	2a	motion < half range	2	motion with gravity eliminated < half range
		2b	motion > half range	3	motion with gravity eliminated > half range
		2c	full motion	4	full motion with gravity eliminated
3	active movement against gravity	3a	motion < half range	5	motion against gravity < half range
		3b	motion > half range	6	motion against gravity > half range
		3c	full motion	7	full motion
4	active movement against resistance	4a	motion < half range		
		4b	motion > half range		
		4c	full motion		
5	normal power and range of motion	5			

Do you think such a hybrid system could be valuable ?

Such a hybrid system combines advantages of both systems.

	1	2	3	4	5	6	7	8	9	
fully disagree	○	○	○	○	○	○	○	○	○	fully agree

I / our clinic would implement this system, given it reaches consensus in the iPluto survey.

	1	2	3	4	5	6	7	8	9	
fully disagree	○	○	○	○	○	○	○	○	○	fully agree

Please provide your comments.

How to score muscles / movements / motion ?

The evaluation of active recovery of muscles / movements is performed differently.

From the first results a possible explanation may be that different joints should be evaluated differently.

Please provide your opinion on the following.
Note: the items below are scored to evaluate **outcome**. (1 year of age and beyond)

For evaluation of **shoulder movements** (abduction, external rotation ...) it is appropriate to express outcome as...

	fully disagree 1	2	3	4	5	6	7	8	fully agree 9
active ROM in degrees									
Active Movement System									
Force (MRC)									
Hybrid system (MRC&AMS)									

For evaluation of **elbow movements** (elbow flexion and extension) it is appropriate to express outcome as...

	fully disagree 1	2	3	4	5	6	7	8	fully agree 9
active ROM in degrees									
Active Movement System									
Force (MRC)									
Hybrid system (MRC&AMS)									

For evaluation of **forearm movements** (pronation, supination) it is appropriate to express outcome as...

	fully disagree 1	2	3	4	5	6	7	8	fully agree 9
active ROM in degrees									
Active Movement System									
Force (MRC)									
Hybrid system (MRC&AMS)									

For evaluation of **wrist movements** (wrist flexion and extension) it is appropriate to express outcome as...

	fully disagree 1	2	3	4	5	6	7	8	fully agree 9
active ROM in degrees									
Active Movement System									
Force (MRC)									
Hybrid system (MRC&AMS)									

For evaluation of **finger movements** (finger flexion, finger extension, thumb) it is appropriate to express outcome as...

	fully disagree 1	2	3	4	5	6	7	8	fully agree 9
active ROM in degrees									
Active Movement System									
Force (MRC)									
Hybrid system (MRC&AMS)									

For evaluation of **evolution of recovery** in the **first year** as a measure to assess lesion severity, it is appropriate to employ...

	fully disagree 1	2	3	4	5	6	7	8	fully agree 9
active ROM in degrees									
Active Movement System									
Force (MRC)									
Hybrid system (MRC&AMS)									

Please provide your comments.

This was the last question.
Thank you very much for your cooperation !

To finish click the submit button.
